# A Re-Evaluation of African Swine Fever Genotypes Based on p72 Sequences Reveals the Existence of Only Six Distinct p72 Groups

**DOI:** 10.3390/v15112246

**Published:** 2023-11-11

**Authors:** Edward Spinard, Mark Dinhobl, Nicolas Tesler, Hillary Birtley, Anthony V. Signore, Aruna Ambagala, Charles Masembe, Manuel V. Borca, Douglas P. Gladue

**Affiliations:** 1 Plum Island Animal Disease Center, Agricultural Research Service, U.S. Department of Agriculture, Foreign Animal Disease Research Unit, Orient, NY 11957, USA; edward.spinard@usda.gov (E.S.); mark.dinhobl@usda.gov (M.D.); nicolastesler2@gmail.com (N.T.); hillary.birtlery@usda.gov (H.B.); 2 National Bio and Agro-Defense Facility, Agricultural Research Service, U.S. Department of Agriculture, Manhattan, KS 66502, USA; 3 Center of Excellence for African Swine Fever Genomics, Guilford, CT 06437, USA; anthony.signore@inspection.gc.ca (A.V.S.); aruna.ambagala@inspection.gc.ca (A.A.); charles.masembe@mak.ac.ug (C.M.); 4 Oak Ridge Institute for Science and Education (ORISE), Oak Ridge, TN 37830, USA; 5 National Centre for Foreign Animal Disease, Canadian Food Inspection Agency, Winnipeg, MB R3E 3M4, Canada; 6 College of Natural Resources (CoNAS), Makerere University, Kampala P.O. Box 7062, Uganda

**Keywords:** African swine fever, ASFV, genotype, p72

## Abstract

The African swine fever virus (ASFV) is currently causing a world-wide pandemic of a highly lethal disease in domestic swine and wild boar. Currently, recombinant ASF live-attenuated vaccines based on a genotype II virus strain are commercially available in Vietnam. With 25 reported ASFV genotypes in the literature, it is important to understand the molecular basis and usefulness of ASFV genotyping, as well as the true significance of genotypes in the epidemiology, transmission, evolution, control, and prevention of ASFV. Historically, genotyping of ASFV was used for the epidemiological tracking of the disease and was based on the analysis of small fragments that represent less than 1% of the viral genome. The predominant method for genotyping ASFV relies on the sequencing of a fragment within the gene encoding the structural p72 protein. Genotype assignment has been accomplished through automated phylogenetic trees or by comparing the target sequence to the most closely related genotyped p72 gene. To evaluate its appropriateness for the classification of genotypes by p72, we reanalyzed all available genomic data for ASFV. We conclude that the majority of p72-based genotypes, when initially created, were neither identified under any specific methodological criteria nor correctly compared with the already existing ASFV genotypes. Based on our analysis of the p72 protein sequences, we propose that the current twenty-five genotypes, created exclusively based on the p72 sequence, should be reduced to only six genotypes. To help differentiate between the new and old genotype classification systems, we propose that Arabic numerals (1, 2, 8, 9, 15, and 23) be used instead of the previously used Roman numerals. Furthermore, we discuss the usefulness of genotyping ASFV isolates based only on the p72 gene sequence.

## 1. Introduction

African swine fever (ASF) is a highly lethal disease of domestic swine and wild boar caused by exposure to the African swine fever virus (ASFV), a large dsDNA virus within the *Asfarviridae* family. The disease, which has been around for just over 100 years [1], is currently causing a global pandemic that started after the introduction of a genotype II strain of ASFV into the republic of Georgia in 2007 [2], which has persistently spread across Europe, Asia, and recently the island of Hispaniola in 2021. Further, derivatives of this strain have also been reported in parts of Africa [3,4]. In recent history, outbreaks of ASFV on domestic or smallholder farms have been restricted to only genotypes I, II, IX, X, XV, and XXIII [2,5,6,7,8]. However, the significance and accuracy of genotypes have become a concern for the ASFV research community. Recently, during the Global Alliance for African Swine Fever (GARA) meeting held in the Dominican Republic in May 2022 and again at the GARA Gap Analysis held in Uganda in February 2023, the significance of p72 genotyping was discussed. ASFV genotyping based on the sequence of p72 was created with the purpose of the epidemiological tracking of different field isolates. However, p72 can remain stable when other parts of the genome undergo significant changes. For example, genotype II strains isolated from extended disease outbreaks [4,7,9] have been observed to contain large genomic deletions, leading to isolates that possess identical p72 sequences but contain genomes with markedly distinct characteristics. Further, reports showing cross-protection between different p72 ASFV genotypes [10,11] suggest that genotyping based on p72 is of limited use in ASFV vaccine matching. Accordingly, the use of p72 genotyping for disease tracking is not as accurate as historical studies would suggest.

Of course, the most accurate classification of ASFV would be based on whole genome sequencing. As of September 2023, there have been 293 full-length ASFV genomes deposited at NCBI; still, most of the full-length genomes originate from isolates collected from outbreaks in Europe and Asia. Accordingly, despite these problems of using p72 to genotype ASFV, the alternative option of using full genomic sequences to classify new isolates presents several constraints in many developing countries facing ASFV outbreaks. These constraints include both the cost of NGS, the availability of reagents, and the absence of dedicated bioinformatic analysis facilities. As a result of these challenges, ASFV genotyping by p72 sequence is the most readily available method, at least until a better and more feasible alternative is provided and widely accepted.

To reevaluate and address the concerns of genotyping ASFV based on p72, we reassessed all the publicly available nucleotide sequences of p72 and reassigned all ASFV isolates into corrected genotypes. We conclude that the majority of p72-based genotypes, when initially created, were neither identified under any specific methodological criteria nor correctly compared with the already existing ASFV genotypes. Some of these errors can easily be explained by honest scientific error or the evolution of more advanced sequencing technologies. We formulated the following criteria to be employed in the formation of the novel genotypes: (1) To assign classification based on potential functional changes, p72 genotyping will be based on the predicted protein sequence. (2) In order to consider the possibility of sequencing errors, each new genotype must include at least one sequence obtained through Next-Generation Sequencing (NGS) or encompass multiple complete sequences from distinct isolates, and (3) a threshold of two amino acid variations, as compared with a validated sequence, was established as the maximum allowable tolerance level. Based on these criteria, we propose that the current ASFV twenty-five genotypes, created exclusively based on the p72 sequence, should be reduced to only six genotypes.

## 2. Materials and Methods

### 2.1. Description of the Dataset

A total of 11,429 ASFV sequences and their corresponding accession numbers originating from both partial and complete ASFV genomes were downloaded from the nucleotide collection of NCBI (https://www.ncbi.nlm.nih.gov/nuccore/) accessed on 15 May 2023, using the search term “African swine fever virus” [porgn: __txid10497] and the Molecule types filter set to “genomic DNA/RNA”. Vaccine strains and recombinant viruses were excluded from analysis. Since growth in primary swine macrophages does not cause any changes to p72 and since most metadata associated with the p72 sequences lacked passage information, unless specifically noted, isolates were not differentiated from lab-type strains.

### 2.2. Data Preprocessing

To extract full-length p72 (646 amino acids) protein sequences from the ASFV nucleotide dataset, open reading frames (ORF) were predicted (Start codon = AUG, minimum length = 30 codons, Genetic code = standard, no open-ended sequences), and amino acid sequences were predicted using CLC Genomics Workbench v. 23 (Qiagen; Hilden, Germany). The amino acid sequences were compared with a curated dataset composed of p72 sequences encoded by Benin 97-1 (AM712239), OURT 88-3 (AM712240), Kenya1950 (AY261360), Malawi Lil-20-1 (AY261361), Mkuzi 1979 (AY261362), Pretorsuskop-96-4 (AY261363), Tengani62 (AY261364), Warmbaths (AY261365), Warthog (AY261366), E75 (FN557520), Ken05-Tk1 (KM111294), Ken06Bus (KM111295), L60 (KM262844), NHV (KM262845), BA71V (U18466), and ASFV-G (LR743116) using BlastP. Protein sequences with high probability matches to the curated p72 database (E value = 0) were retained along with their original accession number and nucleotide sequence (Table S1).

To extract partial p72 (<646 amino acids) protein sequences from the ASFV nucleotide dataset, GFF3 files were downloaded from NCBI using accession numbers that did not contain a full-length p72 sequence. A custom script was used to extract and correctly orientate the nucleotide sequence from accessions that contained the words “p72” or “B646” in the product or name qualifier. The first and last 10 nucleotides were examined for complete or partial sequence hits to the P72-U (5′-GGCACAAGTTCGGACATGT-3′) and P72-D (5′-CTGTGCTGCGTTACAGTAC-3′) sequencing primers. Primer sequences, along with any preceding (P72-U) and proceeding (P72-D) A’s or T’s tails that were added during PCR amplification with Taq DNA polymerase, were trimmed from the partial p72 sequences [12]. The amino acid sequences were then predicted from the trimmed nucleotide sequences (Table S1).

Isolates LIV 5/40 (MN318203), RSA/2/2008 (MN336500), SPEC 57 (MN394630), Zaire (MN630494), RSA/W1/1999 (MN641876), and RSA/2/2004 (MN641877) encoded for extreme variants of p72. Accordingly, raw sequencing data (PRJNA577538, PRJNA577445, PRJNA577546, PRJNA587577, PRJNA587581, and PRJNA587591, respectively) was downloaded and used to correct the p72 sequences.

### 2.3. Data Analysis

DNA and protein alignments were performed in CLC Genomics WorkBench v23 using the following parameters: Gap open cost = 10.0, Gap extension cost = 1.0, End gap cost = Free, Alignment mode = Very accurate (slow), Redo alignments = No, Use fixpoints = No. Pairwise alignment comparison matrices were created from the alignments in CLC Genomics WorkBench.

Counting the number of unique DNA/protein sequences, DNA/protein sequence duplications, and internal matches of partial DNA/protein sequences to longer sequences was performed using custom Python scripts.

To generate phylogenetic trees, representative nucleotide sequences for each historical and proposed new genotype (Tables S2 and S3) were aligned using MAFFT v7.49, totaling 1965 nucleotides in length [13]. Both the nucleotide alignment and predicted amino acid alignment of the same isolates were used to build maximum likelihood phylogenetic trees with IQ-TREE v2.20 [14]. Trees were built under the best-fitting model of nucleotide substitution as determined by ModelFinder. Node support for each tree was assessed by 5000 ultrafast bootstrap replicates [15]. Trees were visualized with the ggtree package in R [16].

## 3. Results and Discussion

### 3.1. P72 Sequences on NCBI

**Figure 1:**
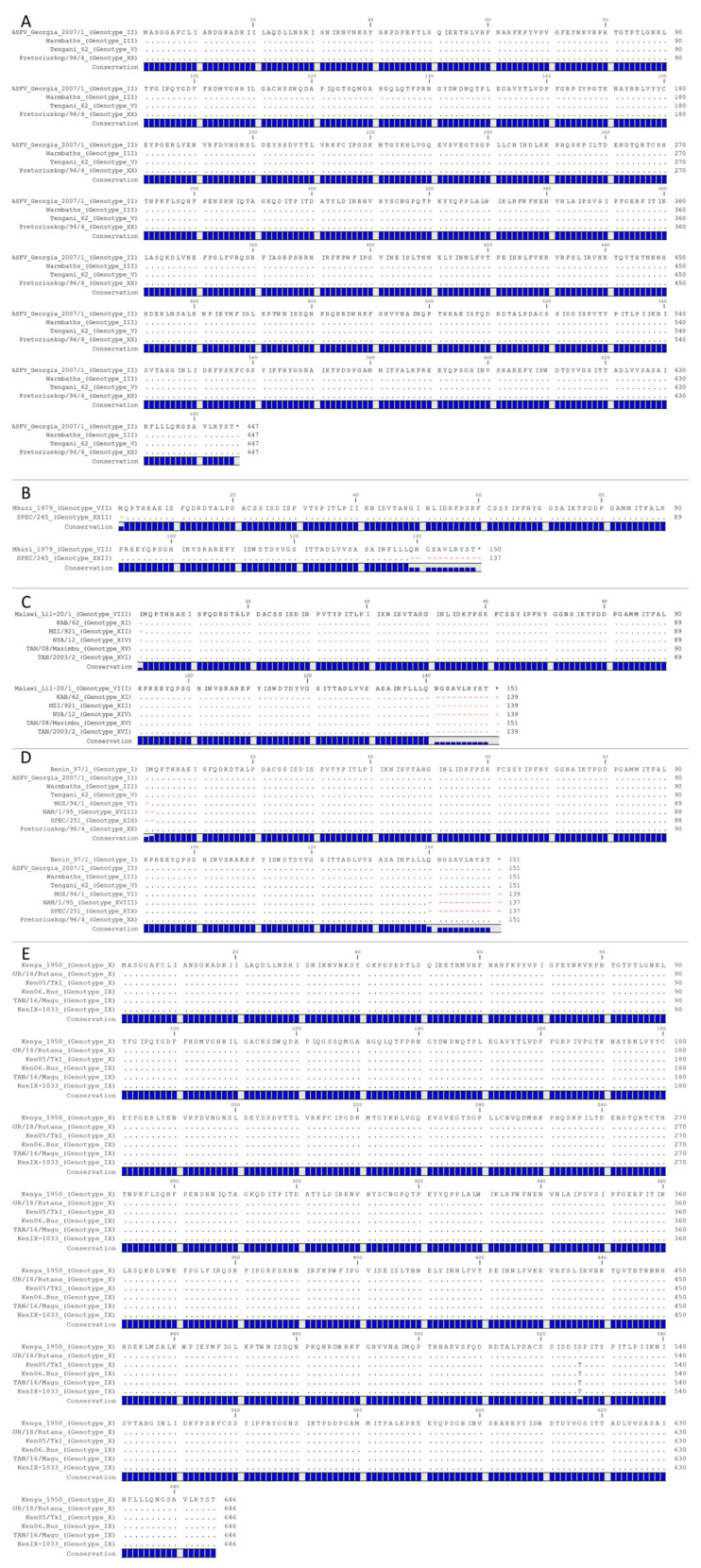
Protein comparison of prototypic p72 (**A**) genotypes: II, III, V, and XX; (**B**) genotypes: VII and XXII; (**C**) genotypes: VIII, XI, XII, XIV, XV, and XVI; (**D**) genotypes: I, II, III, V, VI, VIII, XIX, and XX; (**E**): genotypes IX and X. Matching amino acids are represented by a period, and missing amino acids are represented by a red dash.

All p72 sequences were downloaded from NCBI and processed as described in the Materials and Methods Section (Table S1). When compared by nucleotide sequences, there were 55 uniquely encoded full-length B646L (p72) sequences that had varying degrees of similarity (Table S2). Unless the number of genotypes is expanded to 55, one nucleotide change cannot be the basis for creating a novel p72 genotype. Therefore, it is necessary to establish a specific criterion to govern the classification of p72 sequences and the formation of new virus genotypes. In our opinion, based on the available data, the current methodology utilized for assigning a specific virus to a particular genotype lacks a definitive criterion. Accordingly, we decided to take a closer look at p72 from both the nucleotide and amino acid levels by examining the historic/prototypical p72 sequences that represent the current 25 genotypes (Table S3). While all the nucleotide sequences of p72 are technically unique with the available data, the majority of p72 genotypes have been based on partial sequences that vary in location and length within the p72 ORF (Figure S1). In order to assign classification based on potential functional changes, we decided to continue the analysis on the 25 prototypical p72 sequences by examining the predicted encoded protein sequences (Table S3 and Figure S2). By using this approach, protein sequences of p72 that have been classified into different genotypes were now shown to be exactly matched at the protein level (ASFV-Georgia-2007, Warmbaths, Tengani62, and Pretoriuskop/96/4) (Figure 1A). Further, partial p72 sequences were demonstrated to perfectly match internally to different genotypes that contain a complete p72 sequence (match VII: XXII, match VIII or XV: XI/XII/XIV/XVI, and match I or II or III or V or XX: VI/XVIII/XIX) (Figure 1B–D). Additionally, we discovered a strange occurrence within the genotype X isolate Ken/05/Tk1. Based on nucleotide sequence, Ken/05/Tk1 is similar to other genotype X isolates; however, its predicted amino acid sequence is identical to that of genotype IX isolates (Figure S3 and Figure 1E). Therefore, in its current unregulated state, p72 genotyping should not be considered an appropriate method to classify and differentiate ASFV isolates, as many of the different groups share an exact-matched p72 phenotype (Figure 1 and Figure S2). Additionally, it is imperative to exercise caution when analyzing the sequences, as some p72 sequences within the dataset erroneously included synthetic primer sequences, A/T tails generated from Taq polymerase, and ambiguous nucleotides.

### 3.2. Examination of All the Full-Length p72 Sequences within the p72 ORF in the Database

Protein sequences were predicted from the B646L (p72) coding sequences collected from all ASFV isolates. Within the NCBI database, only 340 of the 2652 sequences encoded a full-length (646 amino acids) p72 sequence (Table S1) and were encoded by 55 unique p72 nucleotide sequences (Table S2). Representative nucleotide sequences were aligned in Supplemental Figure S3. Of the 340 full-length protein sequences, only 23 were unique (Table S4). The number of sequences that are represented by the representative sequence is indicated in Table S4. The 23 unique p72 protein sequences (Table S4) were aligned as seen in Supplemental Figure S4.

### 3.3. Determining Grouping of p72 according to the Amino Acid Sequence

**Figure 2:**
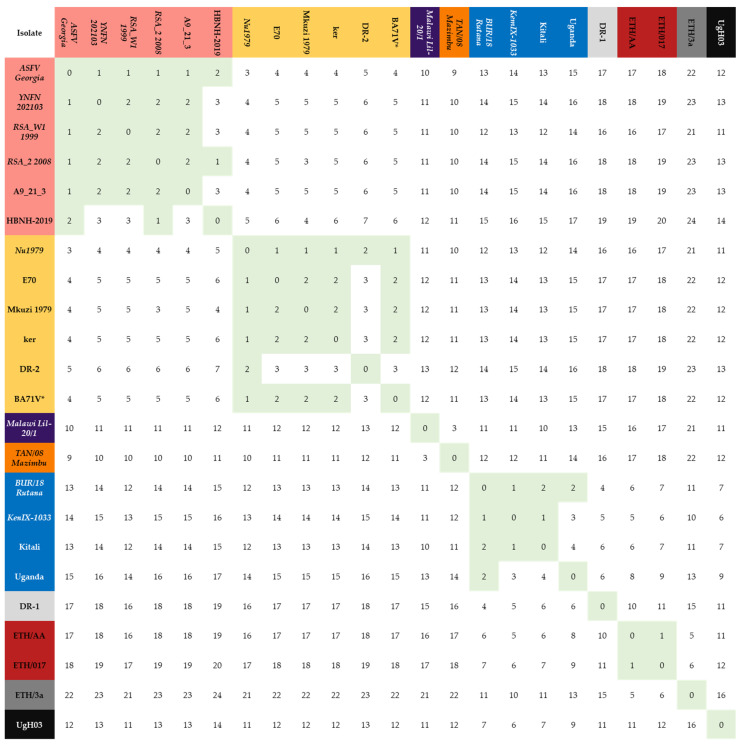
Pairwise comparison demonstrating the number of amino acid differences between the 23 unique full-length p72 sequences. P72 sequences with less than three amino acid changes were highlighted green, and their corresponding isolates were grouped and colored (red through blue) based on likeness. Italicized isolates have been sequenced by NGS.

As previously noted, the establishment of a new genotype has lacked standardization throughout history, posing challenges for laboratories in the classification of certain p72 sequences. Reports have indicated discrepancies in the allowance of two nucleotide changes between different genotypes, with some permitting such changes as LIV/540 (GenBank accession AY351536) vs. LIV/931 (GenBank accession AY351538) and others not historic genotype XI isolate MZI/921 (GenBank accession AY351543) vs. historic genotype XII isolate KAB/62 (GenBank accession AY351522) [17]. This inconsistency has contributed to difficulties in accurately categorizing genotypes. Further evaluation of the dataset has revealed that sequences were deposited at NCBI with sequencing errors. To evaluate a potential standardized criterion at the amino acid level of p72, we performed pairwise comparisons across the 23 unique full-length p72 protein sequences (Figure 2). Naturally, unless the number of genotypes were to decrease from 25 to 23, a degree of tolerance exceeding that of a single amino acid difference would need to be permitted. Accordingly, we developed the following criteria for the establishment of the new genotypes: (1) To account for potential sequencing error, each new genotype must contain at least one representative sequence that was generated via NGS or represents multiple full-length sequences from nonredundant isolates (Table S4) and (2) since the differences between historic genotype I and genotype II isolates have been well established, in order to maintain this separation, a maximum tolerance of two amino acid variations was set as the cutoff. This cutoff separates the 117 sequences represented by the genotype I isolate Nu1979 from the 168 sequences represented by the genotype II isolate ASFV Georgia 2007/01. The p72 sequences encoded by the following isolates were identified as representative sequences: Nu1979 (sequenced via NGS and represents 117 sequences), ASFV Georgia 2007/01 (sequenced via NGS and represents 168 sequences), YNFN202103 (sequenced via NGS and represents zero sequences), RSA_W1_1999 (sequenced via NGS and represents three partial and nineteen full-length sequences), RSA_2_2008 (sequenced via NGS and represents zero sequences), Malawi Lil-20/1 (sequenced via NGS and represents one full-length and two partial sequences), TAN/08/Mazimbu (sequenced via NGS and represents zero full-length and three partial sequences), BUR/18/Rutana (sequenced via NGS and represents eleven full-length and fifty-eight partial sequences), KenIX-1033 (sequenced via NGS and represents ten full-length sequences) and ETH/AA (Sequence via Sanger and represents four full-length sequences).

Using these metrics, we continued our evaluation. The new genotype 2 consists of the representative historic genotype II isolates ASFV Georgia 2007/01 and YNFN202103, the historic genotype IV isolate RSA_W1_1999, and the previously miscategorized genotype XXII isolate RSA_2_2008. Two other variants of ASFV Georgia 2007/01, A9_21_3 and HBNH-2019, also group within the new genotype 2 as they both contain less than three amino acid differences compared with ASFV Georgia 2007/01 and RSA_2_2008. Moreover, as demonstrated in Figure 1, Warmbaths (GenBank accession: AY261365), Tengani 62 (GenBank accession: AY261364), and Pretoriuskop/96/4 (GenBank accession: AY261363), which have been historically identified as genotype III, V, and XX, respectively, were also included in the new genotype 2 since they share an identical p72 protein sequence to ASFV Georgia 2007/01.

We continued examining the remaining isolates using two amino acid changes as the maximum allowance for grouping. New genotype 1 was established using Nu1979 as the representative sequence. Compared with Nu1979 historic genotype I isolates E70, BA71V, DR-2 (GenBank accessions: AM712239, AY578692, M34142, and L76727), the previously ungrouped 1964 isolate from Kerita Kenya, ker (GenBank accession: AY578697), and of the historic genotype VII, Mkuzi 1979 (GenBank accession: AY261362) had a difference of one, one, two, one, and one amino acid(s), respectively (Figure 2) (Table S4). Of note, the p72 protein sequence derived from the complete genome of BA71V (AM712239) is identical to the p72 protein sequence of NU1979, indicating that the BA71V sequence present in GenBank accession M34142 contains amino acid changes unique to that particular laboratory-derived strain (Table S4).

New genotype 8 was composed solely of the representative sequence Malawi Lil-20/1 (AY261361). New genotype 15 was composed solely of the representative sequence TAN/08/Mazimbu (ON409981). The next group, the new genotype 8, combines the historic genotype IX isolate KenIX-1033 and the historic genotype X isolate BUR/18/Rutana (GenBank Accession: MW856067), as there is only one amino acid difference between them. Further, the historic genotype X isolate Uganda (GenBank Accession: L27499) and the previously uncharacterized Kitali (GenBank Accession: MN886937) group within the new genotype 9 as they each contain two amino acid differences compared with BUR/18/Rutana [18].

The next group combined the historic genotype XXIIIa isolate ETH/017 with the representative historic genotype XXIIIb isolate ETH/AA to create the new genotype 23. Interestingly, the other historic genotype XXIIIa isolate, ETH/3a, contained a difference of six amino acids compared with ETH/AA and was classified on its own. The last two groups were occupied solely by DR-1 (GenBank accession: L27498) and UgH03 (GenBank accession: EF121429), respectively. Since the last three groups (DR-1, ETH/3a, and Ugh03) are composed of a single isolate each, we excluded them from the new grouping as the accuracy of the sequencing could not be determined and there was no raw sequencing data or full-length genome to validate that a new genotype exists. However, in the future, these sequences could be validated, and a new genotype could be added based on future studies.

When comparing each distinct p72 sequence to a representative p72 sequence that has been validated by NGS or the existence of multiple identical full-length sequences, employing a threshold of two amino acid differences effectively categorizes the majority of the identified unique p72 sequences. These criteria also allow for non-representative sequences to differ by more than two amino acids. For example, within new genotype 2, HBNH-2019 differs from YNFN202103, RSA_W1_1999, and A9_21_3 by three amino acids. Moreover, within the new genotype 9, Uganda differs from Kitali by four amino acids. It is noteworthy that a representative sequence always deviates from a representative sequence that has been classified into a different genotype by at least four amino acids. Hence, it is imperative to perform the comparisons against a representative sequence to ensure the accuracy and reliability of the results.

**Figure 3:**
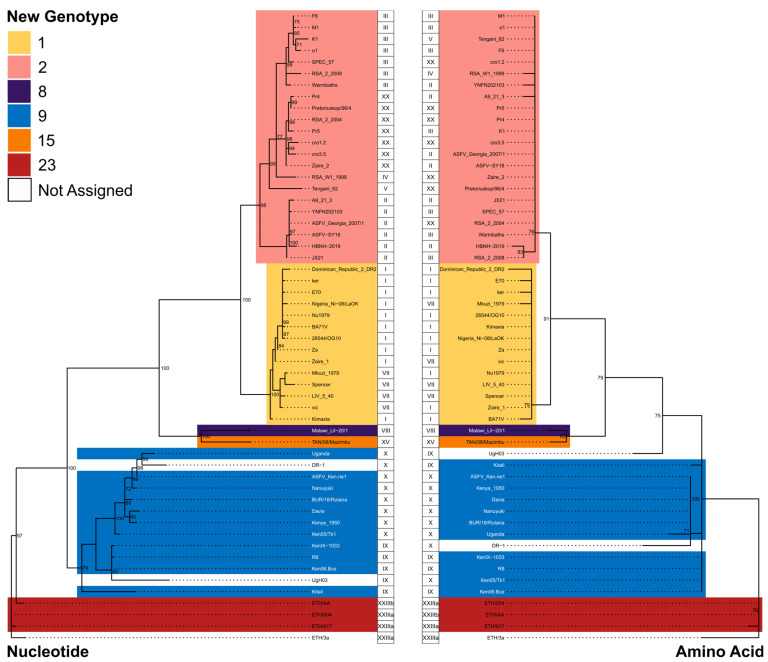
Maximum likelihood phylogenetic trees derived from full-length nucleotide sequence (**left**) and predicted amino acid sequence (**right**) of the African swine fever virus p72 gene. Clades are colored by proposed new genotype designations. Historical genotype designations are displayed next to each tree tip. Internal nodes are annotated with the percentage of bootstrap replicates, resulting in the displayed tree topology. Only bootstrap values > 70 are shown.

Lastly, phylogenetic analysis performed on the 55 full-length (Table S2) and full-length prototypical historical (Table S3) nucleotide and amino acid sequences (Figure 3, Table 1) creates clades that separate in support of our new classification. Interestingly, as previously mentioned, Ken/05/Tk1 groups have historic genotypes X based on nucleotide sequence, but groups have historic genotypes IX based on protein sequence. Both isolates serve as examples that highlight the necessity of utilizing protein sequences rather than nucleotide sequences in order to establish a classification system that is based on the potential functionality of p72. In summary, based on the analysis of full-length p72 protein sequences, all ASFV isolates can be classified into six genotypes instead of the currently accepted twenty-five genotypes.

### 3.4. Analysis of Partial p72 Sequences Available on NCBI

The remaining 2312 partial p72 sequences were compared with the 23 full-length p72 sequences. We observed 222 unique protein sequences encoding for partial p72, of which 110 of them were identical to a portion of one or multiple full-length p72 sequence(s) (Table S1). Of the other one hundred and twelve protein sequences, eight of them (GenBank accessions: AF301545, AF449460, AF449471, AF449475, AF504884, AF504886, AY538726, and FR668420) had ambiguous amino acids and were discarded due to possible sequencing quality issues. The remaining 104 sequences were analyzed, and 77 partial sequences differed by less than three amino acids compared with any of the already established groups. Fifteen of these sequences would fall into one of our newly established groups, and sixty-five of these sequences matched multiple groups. Of the remaining thirty-two partial p72 sequences that differed by three or more unique amino acid sequences compared with their closest match, all but three have been previously assigned to a genotype (Table S5). Since the raw sequencing data is not available, it is possible that many of these unique sequences that were only reported once were due to Sanger sequencing analysis errors, the inability to fully sequence samples from that geographical region, or arose from mixed populations of ASFV in persistently infected species [19]. These partial sequences were not used for any further classification. The isolates, protein sequences, and DNA sequences that are part of the new genotypes are fully described in full in Tables S1, S2 and S4.

### 3.5. ASFV Can Be Classified into Six Genotypes

Table 2 summarizes the consolidation of historical genotypes (historic genotypes based on partial sequences are italicized). The historic genotypes VI, XVIII, and XIX were based on partial sequences that were identical to both genotypes 1 and 2 and, accordingly, were not assigned to any single genotype. Likewise, historic genotypes XI, XII, XIV, and XVI were based on partial sequences that were identical to both genotypes 8 and 15. To our knowledge, we have genotyped every publicly available isolate (Table S2) for future reference. Table 3 shows the number of isolates that compose each new genotype and was calculated from Table S1.

Based on our findings, the new genotype 1 consists of 142 isolates (based on 125 full-length and 17 partial sequences), which are composed of sequences that belong to three of the historic genotypes (I, VII, and XXII). In genotype 1, there are now six full-length unique amino acid sequences that are encoded by 14 different nucleotide sequences.

The new genotype 2 consists of 224 isolates (based on 177 full-length and 47 partial sequences), which are composed of six historic genotypes (II, III, IV, V, XVII, XX, and XXI). In genotype 2, there are six full-length unique amino acid sequences that are encoded by 22 different nucleotide sequences.

The new genotype 8 consists of five isolates (based on three full-length and two partial sequences) representing one historic genotype (VIII). In genotype 8, there is one full-length unique amino acid sequence that is encoded by one nucleotide sequence.

The new genotype 9 consists of 297 isolates (based on 24 full-length and 273 partial sequences) and is composed of two historic genotypes (IX and X). In genotype 9, there are four unique full-length amino acid sequences that are encoded by 11 different nucleotide sequences.

The new genotype 15 consists of four isolates (based on one full-length and three partial sequences) and is composed of one historic genotype (XV). In genotype 9, there is one unique full-length amino acid sequence that is encoded by one nucleotide sequence.

The new genotype 23 consists of ten isolates (based on six full-length and four partial sequences) and is composed of two historic genotypes (XXIIIa and XXIIIb). In genotype 23, there are three full-length unique amino acid sequences that are encoded by three different nucleotide sequences.

Lastly, isolates that fit equally well within multiple genotypes all contained partial sequences. Based on our findings, we can conclude that the old methodology for p72 genotyping without distinct characteristics for defining a new genotype was not the perfect genotyping method, as many of the genotypes that were based on partial sequences can be consolidated into larger genotypes.

## 4. Conclusions

As modern next-generation sequencing technologies can now provide the entire genome sequence of ASFV, classification based on the p72 sequence alone is not an ideal approach for grouping ASFV. However, it is widely used in the literature and is imperative in developing countries where extended outbreaks occur in order to quickly classify ASFV. Further, these regions may not have the ability to perform or analyze next-generation sequencing. However, it is important to note that Sanger sequencing of partial fragments of the p72 gene does not provide any substantial benefits compared with sequencing the entire p72 coding sequence. This is because, even in laboratories where next-generation sequencing of ASFV is not feasible, the feasibility of sequencing the full-length p72 gene is equally attainable as sequencing a fragment of the p72 gene. Further, as our analysis demonstrates, partial sequencing may fail to identify the correct genotype.

Other attempts to group ASFV isolates have relied upon the central variable region (CVR) region of B604L, the E183L gene encoding p54, or EP402R encoding for CD2V. However, we did not consider these sequences in our analysis, as these alternative classification techniques were not performed on all historical isolates. Furthermore, the goal of this analysis was to identify the inherent ambiguities and confusions within the historic p72 genotypes and establish a simplified best-fit criteria for p72 genotyping based on a set of curated p72 protein sequences that will allow for the generation of new p72 genotypes in the future. Still, we acknowledge that any methodology, including our proposed classification, that attempts to classify ASFV strains by using a single or only a few genes would only provide the broadest understanding of disease spread, as changes in other areas of the genome would go unnoticed. For example, the ~6 kb deletion in genotype II recently reported in Nigeria and Ghana [3] would be ignored. Although, in this case, similar phenotypes to Georgia/2007 were reported on these farms and the significance of this deletion is debatable, p72 genotyping alone or in conjunction with p54 and/or CVR sequencing would not allow for differentiation of these strains for disease tracking.

Currently, there is no more relevant methodology to classify ASFV isolates than what is presented in this study. For example, the lack of data regarding the immune response to specific viral proteins correlating with protection makes serotyping all isolates of ASFV a difficult task that will likely require a large effort to test cross-protection in swine against different virulent strains of ASFV. More recent attempts to group ASFV have examined the relationship of multiple Pfam domains and have been used to compare virulent and non-virulent strains [20].

In summary, we report here the classification of ASFV isolates using p72 amino acid sequences and identify six genotypes of ASFV. We believe this is a more modern approach to attempting to group isolates based on a small fragment of the ASFV genome consisting of only one gene. Further, we propose the use of Arabic numerals instead of Roman numerals to differentiate the new classification system from the old. It did not go unnoticed that X and V were interchanged, and isolates were often mislabeled as VII and XII or VIII and XIII. Using the approach of a tolerance of two amino acid changes constituting a new genotype of ASFV, it is in agreement that only historical genotypes I, II, IX, X, XV, and XXIII [2,5,6,7,8] are being reported in recent history and new genotypes 1, 2, 9, 15, and 23, with the only disagreement being that genotype 8, consisting of historical genotypes VIII, XI, XII, XIII, XIV, XV, and XVI has not been reported in recent history. To facilitate easier usage of this new classification system, we have recently developed a free tool for evaluating p72 using two amino acid thresholds, which is available at asfvgenomics.com/upload (accessed on November 1, 2023; manuscript under review).

## Figures and Tables

**Table 1:** Average number of amino acid differences between representative p72 sequences of different genotypes.

	Genotype 2	Genotype 1	Genotype 8	Genotype 9	Genotype 15	Genotype 23
Genotype 2	1.9	4.9	11.0	14.4	10.0	18.2
Genotype 1	4.9	2.0	12.0	13.8	11.0	17.5
Genotype 8	11.0	12.0	0	11.3	3.0	16.5
Genotype 9	14.4	13.8	11.3	2.17	12.3	6.8
Genotype 15	10.0	11.0	3	12.3	0.0	17.5
Genotype 23	18.2	17.5	16.5	6.8	17.5	1.0

**Table 2:** Summary of genotype consolidation.

New Genotype Assignment	Historical Genotype
1	I, VII, and XXII
2	II, III, IV, V, XVII, XX, XXI, and XXIV
8	VIII
9	IX and X
15	XV
23	XXIIIa and XXIIIb

Arabic numerals are used to distinguish the new genotype classification. Genotypes VI, XVIII, and XIX matched genotypes 1 and 2. Genotypes XI, XII, XIV, and XVI matched genotypes 8 and 15. Genotype XIII contained three amino acid differences compared with genotypes 8 and 15.

**Table 3:** Number of isolates that compose each new genotype.

New Genotype	Number of Isolates	Number of Unique Sequences
1	142	18
2	224	34
8	5	3
9	297	30
15	4	2
23	10	5
1 or 2	1840	116
1 or 2 or 8	1	1
1 or 2 or 8 or 9 or 15	1	1
1 or 2 or 8 or 9 or 23	1	1
1 or 2 or 9 or 23	4	1
1 or 8 or 9 or 2 or 15	1	1
1 or 9 or 23	2	1
2 or 9	1	1
8 or 15	72	12
8 or 9 or 15	1	1
9 or 23	31	4
not defined	15	14

Number of isolates includes partial sequences.

## Data Availability

All sequence data was downloaded from NCBI. The GenBank accession number of each sequence has been provided in Table S1.

## Supplementary Materials

The following supporting information can be downloaded at: https://www.mdpi.com/article/10.3390/v15112246/s1, Table S1: Complete list of p72 nucleotide and protein sequences; Table S2: Representative isolates of genotypes and matches (nucleotide); Table S3: Representative isolates of historical genotypes; Table S4: Representative isolates of new genotypes (protein); Table S5: Isolates with partial p72 protein sequences with greater than three amino acid differences; Figure S1: Alignment of historical p72 DNA coding sequences; Figure S2: Alignment of historical p72 protein sequences; Figure S3: Alignment of 55 unique full-length p72 coding DNA sequences; and Figure S4: Alignment of 23 unique full-length p72 protein sequences.
