# Supplementary material for: A Re-Evaluation of African Swine Fever Genotypes Based on p72 Sequences Reveals the Existence of Only Six Distinct p72 Groups
Source: Viruses. 2023 Nov 11;15(11):2246. doi: 10.3390/v15112246 (PMC10675559; doi:10.3390/v15112246)
Supplement: Supplementary file 1 [file viruses-15-02246-s001.zip › Figure S1 Alignment of historical genotypes (nucleotide).pdf]

```
                                                      20                  40                  60                  80
              Benin_97/1_(Genotype_I)  ATGGCATCAG GAGGAGCTTT TTGTCTTATT GCTAACGATG GGAAGGCCGA CAAGATTATA TTGGCCCAAG ACTTGCTTAA TAGCAGGATT 90
     ASFV_Georgia_2007/1_(Genotype_II)  .......... .......... .......... .......... .......... .......... .......... .......G.. .........C 90
             Warmbaths_(Genotype_III)  .......... .......... .......... .......... .......... .......... .......... .......G.. .........C 90
                Warthog_(Genotype_IV)  .......... .......... .......... .......... .......... .......... .......... .......G.. .........C 90
              Tengani_62_(Genotype_V)  .......... .......... .......... .......... .......... T......... .......... .......G.. .........C 90
                MOZ/94/1_(Genotype_VI)  ---------- ---------- ---------- ---------- ---------- ---------- ---------- ---------- ---------- –
             Mkuzi_1979_(Genotype_VII)  .......... .......... .......... .......... .......... .......... .......... .......... .......... 90
      Malawi_Lil-20/1_(Genotype_VIII)  .......... .......... .......... .......... .......... .........C .......... .......... .........C 90
              Ken06.Bus_(Genotype_IX)  .......... .......... .......... .......... .......... ......C..T .......... .......... .........C 90
              Kenya_1950_(Genotype_X)  .......... .......... .......... .......... .......... ......C..T .......... .......... .........C 90
                 KAB/62_(Genotype_XI)  ---------- ---------- ---------- ---------- ---------- ---------- ---------- ---------- ---------- –
               MZI/921_(Genotype_XII)  ---------- ---------- ---------- ---------- ---------- ---------- ---------- ---------- ---------- –
             SUM/1411_(Genotype_XIII)  ---------- ---------- ---------- ---------- ---------- ---------- ---------- ---------- ---------- –
               NYA/12_(Genotype_XIV)  ---------- ---------- ---------- ---------- ---------- ---------- ---------- ---------- ---------- –
          TAN/08/Mazimbu_(Genotype_XV)  .......... .......... .......... .......... .......... .........C .......... .......... .........C 90
            TAN/2003/2_(Genotype_XVI)  ---------- ---------- ---------- ---------- ---------- ---------- ---------- ---------- ---------- –
              ZIM/92/1_(Genotype_XVII)  ---------- ---------- ---------- ---------- ---------- ---------- ---------- ---------- ---------- –
             NAM/1/95_(Genotype_XVIII)  ---------- ---------- ---------- ---------- ---------- ---------- ---------- ---------- ---------- –
              SPEC/251_(Genotype_XIX)  ---------- ---------- ---------- ---------- ---------- ---------- ---------- ---------- ---------- –
     Pretoriuskop/96/4_(Genotype_XX)  .......... .......... .......... .......... .......... .......... .......... .......G.. .........C 90
                RSA/1/96_(Genotype_XXI)  ---------- ---------- ---------- ---------- ---------- ---------- ---------- ---------- ---------- –
             SPEC/245_(Genotype_XXII)  ---------- ---------- ---------- ---------- ---------- ---------- ---------- ---------- ---------- –
           ETH/017_(Genotype_XXIIIa)  .......... .......... .......... ........C. .......... ......C..T .......... .......... .........C 90
             ETH/AA_(Genotype_XXIIIb)  .......... .......... .......... .......... .......... ......C..T .......... .......... .........C 90
           MOZ_10/2006_(Genotype_XXIV)  ---------- ---------- ---------- ---------- ---------- ---------- ---------- ---------- ---------- –
                        Conservation
                                                 100                 120                 140                 160                 180
              Benin_97/1_(Genotype_I)  TCTAACATTA AAAATGTGAA CAAAAGTTAT GGGAAACCCG ACCCCGAACC CACTTTGAGT CAAATCGAAG AAACACATTT GGTTCATTTT 180
     ASFV_Georgia_2007/1_(Genotype_II)  .......... .......... .......... .......... .T........ .......... .......... .......... ...G...... 180
             Warmbaths_(Genotype_III)  .......... .......... .......... .......... .......... .......... .......... .......... ...G...... 180
                Warthog_(Genotype_IV)  .......... .......... .......... .......... .......... .......... .......... .......... ...G...... 180
              Tengani_62_(Genotype_V)  .......... .......... .........C .......... .......... .......... .......... .......... ...G...... 180
                MOZ/94/1_(Genotype_VI)  ---------- ---------- ---------- ---------- ---------- ---------- ---------- ---------- ---------- –
             Mkuzi_1979_(Genotype_VII)  .......... .......... .......... .......... .T........ .......... .......... .......... ...G...... 180
      Malawi_Lil-20/1_(Genotype_VIII)  .......... .......T.. .......... .......... .......... .......... .......... ........A. ...G...... 180
              Ken06.Bus_(Genotype_IX)  .......... .......T.. .......... .......... .......... .......... .......... ........A. ...G...... 180
              Kenya_1950_(Genotype_X)  .......... .......T.. .......... .......... .......... .......... .......... ........A. ...G...... 180
                 KAB/62_(Genotype_XI)  ---------- ---------- ---------- ---------- ---------- ---------- ---------- ---------- ---------- –
               MZI/921_(Genotype_XII)  ---------- ---------- ---------- ---------- ---------- ---------- ---------- ---------- ---------- –
             SUM/1411_(Genotype_XIII)  ---------- ---------- ---------- ---------- ---------- ---------- ---------- ---------- ---------- –
               NYA/12_(Genotype_XIV)  ---------- ---------- ---------- ---------- ---------- ---------- ---------- ---------- ---------- –
          TAN/08/Mazimbu_(Genotype_XV)  .......... .......T.. .......... .......... .T........ .......... .......... ........A. ...G...... 180
            TAN/2003/2_(Genotype_XVI)  ---------- ---------- ---------- ---------- ---------- ---------- ---------- ---------- ---------- –
              ZIM/92/1_(Genotype_XVII)  ---------- ---------- ---------- ---------- ---------- ---------- ---------- ---------- ---------- –
             NAM/1/95_(Genotype_XVIII)  ---------- ---------- ---------- ---------- ---------- ---------- ---------- ---------- ---------- –
              SPEC/251_(Genotype_XIX)  ---------- ---------- ---------- ---------- ---------- ---------- ---------- ---------- ---------- –
     Pretoriuskop/96/4_(Genotype_XX)  .......... .......... .......... ........T. .......... .......... .......... .......... ...G...... 180
                RSA/1/96_(Genotype_XXI)  ---------- ---------- ---------- ---------- ---------- ---------- ---------- ---------- ---------- –
             SPEC/245_(Genotype_XXII)  ---------- ---------- ---------- ---------- ---------- ---------- ---------- ---------- ---------- –
           ETH/017_(Genotype_XXIIIa)  .....T.... .......T.. ...G...... .......... .......... .C........ .......... ........A. ...G...... 180
             ETH/AA_(Genotype_XXIIIb)  .....T.... .......T.. ...G...... .......... .......... .......... .......... ........A. ...G...... 180
           MOZ_10/2006_(Genotype_XXIV)  ---------- ---------- ---------- ---------- ---------- ---------- ---------- ---------- ---------- –
                        Conservation
                                                      200                 220                 240                 260
              Benin_97/1_(Genotype_I)  AATGCGCATT TTAAGCCTTA TGTTCCAGTA GGGTTTGAAT ACAATAAAGT ACGCCCGCAT ACGGGTACCC CCACCTTGGG AAACAAGCTT 270
     ASFV_Georgia_2007/1_(Genotype_II)  .......... .......... .......... .......... .......... .......... .......... .......... .......... 270
             Warmbaths_(Genotype_III)  .......... .......... ......G... .......... .......... .......... .......... .......... .......... 270
                Warthog_(Genotype_IV)  .......... .......... .......... .......... .......... .......... .......... .......... .......... 270
              Tengani_62_(Genotype_V)  .......... .......... .......... .......... .......... .......... .......... .......... .......... 270
                MOZ/94/1_(Genotype_VI)  ---------- ---------- ---------- ---------- ---------- ---------- ---------- ---------- ---------- –
             Mkuzi_1979_(Genotype_VII)  .......... .......... .......... .......... .......... .......... .......... .......... .......... 270
      Malawi_Lil-20/1_(Genotype_VIII)  .....A.... .......... .......A.. .......... .......... G..T...... .......... ....A..... C.....A... 270
              Ken06.Bus_(Genotype_IX)  ..C..A.... .......... C..C...A.. .......... .......... ...T..A... .......... ....A..... C......... 270
              Kenya_1950_(Genotype_X)  ..C..A.... .......... ...C...A.. .......... .......... ...T..A... .......... ....A..... C......... 270
                 KAB/62_(Genotype_XI)  ---------- ---------- ---------- ---------- ---------- ---------- ---------- ---------- ---------- –
               MZI/921_(Genotype_XII)  ---------- ---------- ---------- ---------- ---------- ---------- ---------- ---------- ---------- –
             SUM/1411_(Genotype_XIII)  ---------- ---------- ---------- ---------- ---------- ---------- ---------- ---------- ---------- –
               NYA/12_(Genotype_XIV)  ---------- ---------- ---------- ---------- ---------- ---------- ---------- ---------- ---------- –
          TAN/08/Mazimbu_(Genotype_XV)  .....A.... .......... C......... ..A....... .......... ...T...... .......... ....A..... C......... 270
            TAN/2003/2_(Genotype_XVI)  ---------- ---------- ---------- ---------- ---------- ---------- ---------- ---------- ---------- –
              ZIM/92/1_(Genotype_XVII)  ---------- ---------- ---------- ---------- ---------- ---------- ---------- ---------- ---------- –
             NAM/1/95_(Genotype_XVIII)  ---------- ---------- ---------- ---------- ---------- ---------- ---------- ---------- ---------- –
              SPEC/251_(Genotype_XIX)  ---------- ---------- ---------- ---------- ---------- ---------- ---------- ---------- ---------- –
     Pretoriuskop/96/4_(Genotype_XX)  .......... .......... .......... .......... .......... .......... .......... .......... .......... 270
                RSA/1/96_(Genotype_XXI)  ---------- ---------- ---------- ---------- ---------- ---------- ---------- ---------- ---------- –
             SPEC/245_(Genotype_XXII)  ---------- ---------- ---------- ---------- ---------- ---------- ---------- ---------- ---------- –
           ETH/017_(Genotype_XXIIIa)  ..C..A.... .......... ...C...A.. .......... .......... ...T..A... .......... ....A..... C......... 270
             ETH/AA_(Genotype_XXIIIb)  ..C..A.... .......... ...C...A.. .......... .......... ...T..A... .......... ....A..... C......... 270
           MOZ_10/2006_(Genotype_XXIV)  ---------- ---------- ---------- ---------- ---------- ---------- ---------- ---------- ---------- –
                        Conservation
```

```
                                    280                 300                 320                 340                 360
Benin_97/1_(Genotype_I)              ACCTTTGGTA TTCCCCAGTA CGGAGACTTT TTCCATGATA TGGTGGGCCA CCATATATTG GGTGCATGTC ATTCGTCCTG GCAGGATGCT 360
ASFV_Georgia_2007/1_(Genotype_II)    .......... .......... .......... .......... .......... T......... .......... ....A..... .......... 360
Warmbaths_(Genotype_III)             .......... .......... .......... .......... .......... T......... .......... .......... .......... 360
Warthog_(Genotype_IV)                .......... .......... .......... .......... .......... T......... .......... .......... .......... 360
Tengani_62_(Genotype_V)              .......... .......... .......... .......... .......... T......... .......... .......... .......... 360
MOZ/94/1_(Genotype_VI)               ---------- ---------- ---------- ---------- ---------- ---------- ---------- ---------- ---------- -
Mkuzi_1979_(Genotype_VII)            .......... .......... .......... .......... .......... .......... .......... .......... .......... 360
Malawi_Lil-20/1_(Genotype_VIII)      ..T....... ....T..... .......... .......... ....A..... ....G..... .......... ....T..... .......... 360
Ken06.Bus_(Genotype_IX)              .......... .......... .......... .......... ....A..... .......... ..C..G.... ....C..... .......... 360
Kenya_1950_(Genotype_X)              .......... .......... .......... .......... ....A..... .......... .....G.... ....C..... .......... 360
KAB/62_(Genotype_XI)                 ---------- ---------- ---------- ---------- ---------- ---------- ---------- ---------- ---------- -
MZI/921_(Genotype_XII)               ---------- ---------- ---------- ---------- ---------- ---------- ---------- ---------- ---------- -
SUM/1411_(Genotype_XIII)             ---------- ---------- ---------- ---------- ---------- ---------- ---------- ---------- ---------- -
NYA/12_(Genotype_XIV)                ---------- ---------- ---------- ---------- ---------- ---------- ---------- ---------- ---------- -
TAN/08/Mazimbu_(Genotype_XV)         ..T....... .......... T......... .......... ....A..... .......... .......... ....C..... .......... 360
TAN/2003/2_(Genotype_XVI)            ---------- ---------- ---------- ---------- ---------- ---------- ---------- ---------- ---------- -
ZIM/92/1_(Genotype_XVII)             ---------- ---------- ---------- ---------- ---------- ---------- ---------- ---------- ---------- -
NAM/1/95_(Genotype_XVIII)            ---------- ---------- ---------- ---------- ---------- ---------- ---------- ---------- ---------- -
SPEC/251_(Genotype_XIX)              ---------- ---------- ---------- ---------- ---------- ---------- ---------- ---------- ---------- -
Pretoriuskop/96/4_(Genotype_XX)      .......... .......... .......... .......... .......... T......... .......... .......... .......... 360
RSA/1/96_(Genotype_XXI)              ---------- ---------- ---------- ---------- ---------- ---------- ---------- ---------- ---------- -
SPEC/245_(Genotype_XXII)             ---------- ---------- ---------- ---------- ---------- ---------- ---------- ---------- ---------- -
ETH/017_(Genotype_XXIIIa)            .......... .......... .......... .......... ....A..... .......... ..C....... ....C..... .......... 360
ETH/AA_(Genotype_XXIIIb)             .......... .......... .......... .......... ....A..... .......... ..C....... ....C..... .......... 360
MOZ_10/2006_(Genotype_XXIV)          ---------- ---------- ---------- ---------- ---------- ---------- ---------- ---------- ---------- -
Conservation

                                                  380                 400                 420                 440
Benin_97/1_(Genotype_I)              CCGATTCAGG GCACGGCCCA GATGGGGGCC CATGGTCAGC TTCAAACGTT TCCTCGCAAC GGATATGACT GGGACAACCA AACACCTTTA 450
ASFV_Georgia_2007/1_(Genotype_II)    .......... .....T.... .......... .....G.... .......... .......... .......... .......... ......C... 450
Warmbaths_(Genotype_III)             .......... ....AT.... .......... ..C..G.... .......... .......... .......... .......... .......... 450
Warthog_(Genotype_IV)                .......... .....T.... .......... .....G.... .......... .......... .......... .......... .......... 450
Tengani_62_(Genotype_V)              .......... .....T.... .......... .....G.... .......... .......... .......... .......... .......... 450
MOZ/94/1_(Genotype_VI)               ---------- ---------- ---------- ---------- ---------- ---------- ---------- ---------- ---------- -
Mkuzi_1979_(Genotype_VII)            .......... .......... .......... .......... .......... .......... .......... .......... .......... 450
Malawi_Lil-20/1_(Genotype_VIII)      .......... ..T.AT.... .......... .......... .......... .......... .......... .......... .......... 450
Ken06.Bus_(Genotype_IX)              .......... ..T.AT.... .......... .......... .......... .......... .......... .......... .........G 450
Kenya_1950_(Genotype_X)              .......... ..T.AT.... .......... .......... .......... .......... .......... .......... .........G 450
KAB/62_(Genotype_XI)                 ---------- ---------- ---------- ---------- ---------- ---------- ---------- ---------- ---------- -
MZI/921_(Genotype_XII)               ---------- ---------- ---------- ---------- ---------- ---------- ---------- ---------- ---------- -
SUM/1411_(Genotype_XIII)             ---------- ---------- ---------- ---------- ---------- ---------- ---------- ---------- ---------- -
NYA/12_(Genotype_XIV)                ---------- ---------- ---------- ---------- ---------- ---------- ---------- ---------- ---------- -
TAN/08/Mazimbu_(Genotype_XV)         .......... ..T.AT.... ......A... .......... .......... .......... .......... .......... .......... 450
TAN/2003/2_(Genotype_XVI)            ---------- ---------- ---------- ---------- ---------- ---------- ---------- ---------- ---------- -
ZIM/92/1_(Genotype_XVII)             ---------- ---------- ---------- ---------- ---------- ---------- ---------- ---------- ---------- -
NAM/1/95_(Genotype_XVIII)            ---------- ---------- ---------- ---------- ---------- ---------- ---------- ---------- ---------- -
SPEC/251_(Genotype_XIX)              ---------- ---------- ---------- ---------- ---------- ---------- ---------- ---------- ---------- -
Pretoriuskop/96/4_(Genotype_XX)      .......... .....T.... .......... .....G.... .......... .......... .......... .......... .......... 450
RSA/1/96_(Genotype_XXI)              ---------- ---------- ---------- ---------- ---------- ---------- ---------- ---------- ---------- -
SPEC/245_(Genotype_XXII)             ---------- ---------- ---------- ---------- ---------- ---------- ---------- ---------- ---------- -
ETH/017_(Genotype_XXIIIa)            .....A.... ....AT.... .......... .......G.. .A........ .......... .......... .......... ......C..G 450
ETH/AA_(Genotype_XXIIIb)             .....A.... ....AT.... .......... .......G.. .A........ .......... .......... .......... ......C..G 450
MOZ_10/2006_(Genotype_XXIV)          ---------- ---------- ---------- ---------- ---------- ---------- ---------- ---------- ---------- -
Conservation

                                    460                 480                 500                 520                 540
Benin_97/1_(Genotype_I)              GAGGGCGCCG TTTACACGCT TGTAGATCCC TTTGGAAGAC CTATTGTACC CGGCACAAAG AATGCGTACC GAAACTTGGT TTACTACTGC 540
ASFV_Georgia_2007/1_(Genotype_II)    .......... .......... .........T .......... .C........ .......... .......... .......... .......... 540
Warmbaths_(Genotype_III)             .......... .......... .........T .......... .C........ .......... .......... .......... .......... 540
Warthog_(Genotype_IV)                .......... .......... .........T .......... .C........ .......... .......... .......... .......... 540
Tengani_62_(Genotype_V)              .......... .......... .........T .......... .C........ .......... .....A.... ....T..... .......... 540
MOZ/94/1_(Genotype_VI)               ---------- ---------- ---------- ---------- ---------- ---------- ---------- ---------- ---------- -
Mkuzi_1979_(Genotype_VII)            .......... .......... .......... .......... .......... .......... .......... .......... .......... 540
Malawi_Lil-20/1_(Genotype_VIII)      ..A....... .......... ...G.....T .....G.... .C.....C.. .......... .......... .......... .......... 540
Ken06.Bus_(Genotype_IX)              .......... .......... ...G.....T .....G..G. .C.....G.. .......... .......... .......... .......... 540
Kenya_1950_(Genotype_X)              .......... .......... ...G.....T .....G..G. .C........ .......... .......... .......... .......... 540
KAB/62_(Genotype_XI)                 ---------- ---------- ---------- ---------- ---------- ---------- ---------- ---------- ---------- -
MZI/921_(Genotype_XII)               ---------- ---------- ---------- ---------- ---------- ---------- ---------- ---------- ---------- -
SUM/1411_(Genotype_XIII)             ---------- ---------- ---------- ---------- ---------- ---------- ---------- ---------- ---------- -
NYA/12_(Genotype_XIV)                ---------- ---------- ---------- ---------- ---------- ---------- ---------- ---------- ---------- -
TAN/08/Mazimbu_(Genotype_XV)         ..A....... .......... ...G.....T .......... .C........ T......... .......... .......... .......... 540
TAN/2003/2_(Genotype_XVI)            ---------- ---------- ---------- ---------- ---------- ---------- ---------- ---------- ---------- -
ZIM/92/1_(Genotype_XVII)             ---------- ---------- ---------- ---------- ---------- ---------- ---------- ---------- ---------- -
NAM/1/95_(Genotype_XVIII)            ---------- ---------- ---------- ---------- ---------- ---------- ---------- ---------- ---------- -
SPEC/251_(Genotype_XIX)              ---------- ---------- ---------- ---------- ---------- ---------- ---------- ---------- ---------- -
Pretoriuskop/96/4_(Genotype_XX)      .......... .......... .........T .......... .C........ .......... .......... .......... .......... 540
RSA/1/96_(Genotype_XXI)              ---------- ---------- ---------- ---------- ---------- ---------- ---------- ---------- ---------- -
SPEC/245_(Genotype_XXII)             ---------- ---------- ---------- ---------- ---------- ---------- ---------- ---------- ---------- -
ETH/017_(Genotype_XXIIIa)            .......... .......... ...G.....T .....G.... .C........ .......... .......... .......... .......... 540
ETH/AA_(Genotype_XXIIIb)             .......... .......... ...G.....T .....G.... .C........ .......... .......... .......... .......... 540
MOZ_10/2006_(Genotype_XXIV)          ---------- ---------- ---------- ---------- ---------- ---------- ---------- ---------- ---------- -
Conservation
```

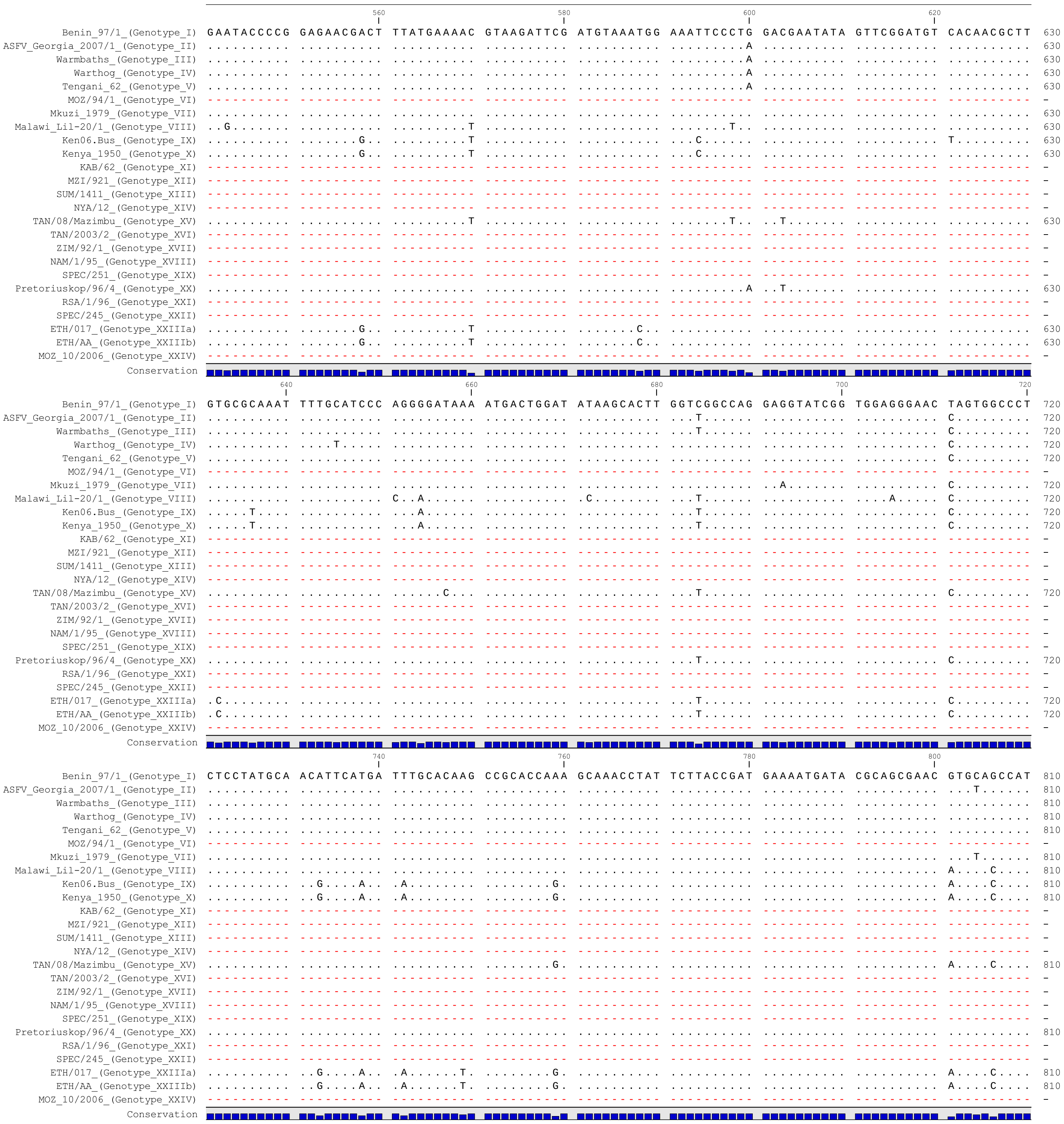

560 580 600 620
Benin_97/1_(Genotype_I) GAATACCCCG GAGAACGACT TTATGAAAAC GTAAGATTCG ATGTAAATGG AAATTCCCTG GACGAATATA GTTCGGATGT CACAACGCTT 630
ASFV_Georgia_2007/1_(Genotype_II) ....A 630
Warmbaths_(Genotype_III) ....A 630
Warthog_(Genotype_IV) ....A 630
Tengani_62_(Genotype_V) ....A 630
MOZ/94/1_(Genotype_VI) ---------- ---------- ---------- ---------- ---------- ---------- ---------- ---------- ---------- -
Mkuzi_1979_(Genotype_VII) 630
Malawi_Lil-20/1_(Genotype_VIII) ..G....... ....T ....T.. 630
Ken06.Bus_(Genotype_IX) ....G.. ....T ....C...... .... T......... 630
Kenya_1950_(Genotype_X) ....G.. ....T ....C...... 630
KAB/62_(Genotype_XI) ---------- ---------- ---------- ---------- ---------- ---------- ---------- ---------- ---------- -
MZI/921_(Genotype_XII) ---------- ---------- ---------- ---------- ---------- ---------- ---------- ---------- ---------- -
SUM/1411_(Genotype_XIII) ---------- ---------- ---------- ---------- ---------- ---------- ---------- ---------- ---------- -
NYA/12_(Genotype_XIV) ---------- ---------- ---------- ---------- ---------- ---------- ---------- ---------- ---------- -
TAN/08/Mazimbu_(Genotype_XV) ....T ....T.. ..T....... 630
TAN/2003/2_(Genotype_XVI) ---------- ---------- ---------- ---------- ---------- ---------- ---------- ---------- ---------- -
ZIM/92/1_(Genotype_XVII) ---------- ---------- ---------- ---------- ---------- ---------- ---------- ---------- ---------- -
NAM/1/95_(Genotype_XVIII) ---------- ---------- ---------- ---------- ---------- ---------- ---------- ---------- ---------- -
SPEC/251_(Genotype_XIX) ---------- ---------- ---------- ---------- ---------- ---------- ---------- ---------- ---------- -
Pretoriuskop/96/4_(Genotype_XX) ....A ..T....... 630
RSA/1/96_(Genotype_XXI) ---------- ---------- ---------- ---------- ---------- ---------- ---------- ---------- ---------- -
SPEC/245_(Genotype_XXII) ---------- ---------- ---------- ---------- ---------- ---------- ---------- ---------- ---------- -
ETH/017_(Genotype_XXIIIa) ....G.. ....T ....C.. 630
ETH/AA_(Genotype_XXIIIb) ....G.. ....T ....C.. 630
MOZ_10/2006_(Genotype_XXIV) ---------- ---------- ---------- ---------- ---------- ---------- ---------- ---------- ---------- -
Conservation
640 660 680 700 720
Benin_97/1_(Genotype_I) GTGCGCAAAT TTTGCATCCC AGGGGATAAA ATGACTGGAT ATAAGCACTT GGTCGGCCAG GAGGTATCGG TGGAGGGAAC TAGTGGCCCT 720
ASFV_Georgia_2007/1_(Genotype_II) ....T...... .... C......... 720
Warmbaths_(Genotype_III) ....T...... .... C......... 720
Warthog_(Genotype_IV) ....T..... .... C......... 720
Tengani_62_(Genotype_V) .... C......... 720
MOZ/94/1_(Genotype_VI) ---------- ---------- ---------- ---------- ---------- ---------- ---------- ---------- ---------- -
Mkuzi_1979_(Genotype_VII) ....A....... .... C......... 720
Malawi_Lil-20/1_(Genotype_VIII) .... C..A...... .... .C........ ...T...... ....A..... C......... 720
Ken06.Bus_(Genotype_IX) .....T.... ....A...... ....T...... .... C......... 720
Kenya_1950_(Genotype_X) .....T.... ....A...... ....T...... .... C......... 720
KAB/62_(Genotype_XI) ---------- ---------- ---------- ---------- ---------- ---------- ---------- ---------- ---------- -
MZI/921_(Genotype_XII) ---------- ---------- ---------- ---------- ---------- ---------- ---------- ---------- ---------- -
SUM/1411_(Genotype_XIII) ---------- ---------- ---------- ---------- ---------- ---------- ---------- ---------- ---------- -
NYA/12_(Genotype_XIV) ---------- ---------- ---------- ---------- ---------- ---------- ---------- ---------- ---------- -
TAN/08/Mazimbu_(Genotype_XV) ....C... ....T...... .... C......... 720
TAN/2003/2_(Genotype_XVI) ---------- ---------- ---------- ---------- ---------- ---------- ---------- ---------- ---------- -
ZIM/92/1_(Genotype_XVII) ---------- ---------- ---------- ---------- ---------- ---------- ---------- ---------- ---------- -
NAM/1/95_(Genotype_XVIII) ---------- ---------- ---------- ---------- ---------- ---------- ---------- ---------- ---------- -
SPEC/251_(Genotype_XIX) ---------- ---------- ---------- ---------- ---------- ---------- ---------- ---------- ---------- -
Pretoriuskop/96/4_(Genotype_XX) ....T...... .... C......... 720
RSA/1/96_(Genotype_XXI) ---------- ---------- ---------- ---------- ---------- ---------- ---------- ---------- ---------- -
SPEC/245_(Genotype_XXII) ---------- ---------- ---------- ---------- ---------- ---------- ---------- ---------- ---------- -
ETH/017_(Genotype_XXIIIa) .C........ ....T...... .... C......... 720
ETH/AA_(Genotype_XXIIIb) .C........ ....T...... .... C......... 720
MOZ_10/2006_(Genotype_XXIV) ---------- ---------- ---------- ---------- ---------- ---------- ---------- ---------- ---------- -
Conservation
740 760 780 800
Benin_97/1_(Genotype_I) CTCCTATGCA ACATTCATGA TTTGCACAAG CCGCACCAAA GCAAACCTAT TCTTACCGAT GAAAATGATA CGCAGCGAAC GTGCAGCCAT 810
ASFV_Georgia_2007/1_(Genotype_II) ....T...... 810
Warmbaths_(Genotype_III) 810
Warthog_(Genotype_IV) 810
Tengani_62_(Genotype_V) 810
MOZ/94/1_(Genotype_VI) ---------- ---------- ---------- ---------- ---------- ---------- ---------- ---------- ---------- -
Mkuzi_1979_(Genotype_VII) ....T...... 810
Malawi_Lil-20/1_(Genotype_VIII) .... A....C.... 810
Ken06.Bus_(Genotype_IX) ....G....A.. .A........ ....G. .... A....C.... 810
Kenya_1950_(Genotype_X) ....G....A.. .A........ ....G. .... A....C.... 810
KAB/62_(Genotype_XI) ---------- ---------- ---------- ---------- ---------- ---------- ---------- ---------- ---------- -
MZI/921_(Genotype_XII) ---------- ---------- ---------- ---------- ---------- ---------- ---------- ---------- ---------- -
SUM/1411_(Genotype_XIII) ---------- ---------- ---------- ---------- ---------- ---------- ---------- ---------- ---------- -
NYA/12_(Genotype_XIV) ---------- ---------- ---------- ---------- ---------- ---------- ---------- ---------- ---------- -
TAN/08/Mazimbu_(Genotype_XV) ....G. .... A....C.... 810
TAN/2003/2_(Genotype_XVI) ---------- ---------- ---------- ---------- ---------- ---------- ---------- ---------- ---------- -
ZIM/92/1_(Genotype_XVII) ---------- ---------- ---------- ---------- ---------- ---------- ---------- ---------- ---------- -
NAM/1/95_(Genotype_XVIII) ---------- ---------- ---------- ---------- ---------- ---------- ---------- ---------- ---------- -
SPEC/251_(Genotype_XIX) ---------- ---------- ---------- ---------- ---------- ---------- ---------- ---------- ---------- -
Pretoriuskop/96/4_(Genotype_XX) 810
RSA/1/96_(Genotype_XXI) ---------- ---------- ---------- ---------- ---------- ---------- ---------- ---------- ---------- -
SPEC/245_(Genotype_XXII) ---------- ---------- ---------- ---------- ---------- ---------- ---------- ---------- ---------- -
ETH/017_(Genotype_XXIIIa) ....G....A.. .A......T. ....G. .... A....C.... 810
ETH/AA_(Genotype_XXIIIb) ....G....A.. .A......T. ....G. .... A....C.... 810
MOZ_10/2006_(Genotype_XXIV) ---------- ---------- ---------- ---------- ---------- ---------- ---------- ---------- ---------- -
Conservation

```
                                  820        840        860        880        900
Benin_97/1_(Genotype_I)           ACCAACCCGA AATTCCTTTC ACAACATTTT CCCGAGAACT CTCACAATAT CCAAACAGCA GGTAAACAAG ATATTACTCC TATTACGGAC 900
ASFV_Georgia_2007/1_(Genotype_II) .......... ....T..... ...G...... .......... .......... .......... .......... .......... ...C...... 900
Warmbaths_(Genotype_III)          .......... ....T..... .......... .......... .......... .......... .......... ....C..... ...C...... 900
Warthog_(Genotype_IV)             .......... ....T..... .......... .......... .......... .......... .......... ....C..... ...C...... 900
Tengani_62_(Genotype_V)           .....T.... ....T..... ...G...... .......... ....T..... .......... .......... ....C..... ...C...... 900
MOZ/94/1_(Genotype_VI)            ---------- ---------- ---------- ---------- ---------- ---------- ---------- ---------- ---------- -
Mkuzi_1979_(Genotype_VII)         .......... .......... .......... .......... .......... .......... .......... .......... ...C...... 900
Malawi_Lil-20/1_(Genotype_VIII)   .......... ....T..... ...G...... .......... .C..T..... .......... .......... .......... .........T 900
Ken06.Bus_(Genotype_IX)           .......... ....T..... ...G...... .......... .C........ .......... .......... .......C.. .......... 900
Kenya_1950_(Genotype_X)           .......... ....T..... ...G...... .......... .C........ .......... .......... .......C.. .......... 900
KAB/62_(Genotype_XI)              ---------- ---------- ---------- ---------- ---------- ---------- ---------- ---------- ---------- -
MZI/921_(Genotype_XII)            ---------- ---------- ---------- ---------- ---------- ---------- ---------- ---------- ---------- -
SUM/1411_(Genotype_XIII)          ---------- ---------- ---------- ---------- ---------- ---------- ---------- ---------- ---------- -
NYA/12_(Genotype_XIV)             ---------- ---------- ---------- ---------- ---------- ---------- ---------- ---------- ---------- -
TAN/08/Mazimbu_(Genotype_XV)      .......... ....T..... ...G...... .......... ....T..... .......... .......... .......... .........T 900
TAN/2003/2_(Genotype_XVI)         ---------- ---------- ---------- ---------- ---------- ---------- ---------- ---------- ---------- -
ZIM/92/1_(Genotype_XVII)          ---------- ---------- ---------- ---------- ---------- ---------- ---------- ---------- ---------- -
NAM/1/95_(Genotype_XVIII)         ---------- ---------- ---------- ---------- ---------- ---------- ---------- ---------- ---------- -
SPEC/251_(Genotype_XIX)           ---------- ---------- ---------- ---------- ---------- ---------- ---------- ---------- ---------- -
Pretoriuskop/96/4_(Genotype_XX)   .......... ....T..... .......... .......... .......... .......... .......... ....C..... ...C...... 900
RSA/1/96_(Genotype_XXI)           ---------- ---------- ---------- ---------- ---------- ---------- ---------- ---------- ---------- -
SPEC/245_(Genotype_XXII)          ---------- ---------- ---------- ---------- ---------- ---------- ---------- ---------- ---------- -
ETH/017_(Genotype_XXIIIa)         .......... ....T..... ...G...... .......... .C........ .........G .......... .......C.. .......... 900
ETH/AA_(Genotype_XXIIIb)          .......... ....T..... ...G...... .......... .C........ .........G .......... .......C.. .......... 900
MOZ_10/2006_(Genotype_XXIV)       ---------- ---------- ---------- ---------- ---------- ---------- ---------- ---------- ---------- -
Conservation

                                             920        940        960        980
Benin_97/1_(Genotype_I)           GCAACGTATC TGGACATAAG ACGTAATGTT CATTACAGCT GTAATGGACC TCAAACCCCT AAATACTATC AGCCCCCTCT TGCGCTCTGG 990
ASFV_Georgia_2007/1_(Genotype_II) .......... .......... .......... .......... .......... .......... .......... .......... .......... 990
Warmbaths_(Genotype_III)          .......... .......... .......... .......... .......... .......... .......... .......... .......... 990
Warthog_(Genotype_IV)             .......... .......... .......... .......... .......... .......... .......... .......... .......... 990
Tengani_62_(Genotype_V)           .......... .......... .......... .......... .......... .......... .......... .......... .......... 990
MOZ/94/1_(Genotype_VI)            ---------- ---------- ---------- ---------- ---------- ---------- ---------- ---------- ---------- -
Mkuzi_1979_(Genotype_VII)         .......... .......... .......... .......... .......... .......... .......... .......... .......... 990
Malawi_Lil-20/1_(Genotype_VIII)   A......... .......... .......... ..A....... .......C.. .......... .......... .......... .......... 990
Ken06.Bus_(Genotype_IX)           .......... .......... .......... .......... .......... .......... .......... .......... .......... 990
Kenya_1950_(Genotype_X)           .......... .......... .......... .......... .......... .......... .......... .......... .......... 990
KAB/62_(Genotype_XI)              ---------- ---------- ---------- ---------- ---------- ---------- ---------- ---------- ---------- -
MZI/921_(Genotype_XII)            ---------- ---------- ---------- ---------- ---------- ---------- ---------- ---------- ---------- -
SUM/1411_(Genotype_XIII)          ---------- ---------- ---------- ---------- ---------- ---------- ---------- ---------- ---------- -
NYA/12_(Genotype_XIV)             ---------- ---------- ---------- ---------- ---------- ---------- ---------- ---------- ---------- -
TAN/08/Mazimbu_(Genotype_XV)      A......... .......... .......... ..A....... .......C.. .......... .......... .......... .......... 990
TAN/2003/2_(Genotype_XVI)         ---------- ---------- ---------- ---------- ---------- ---------- ---------- ---------- ---------- -
ZIM/92/1_(Genotype_XVII)          ---------- ---------- ---------- ---------- ---------- ---------- ---------- ---------- ---------- -
NAM/1/95_(Genotype_XVIII)         ---------- ---------- ---------- ---------- ---------- ---------- ---------- ---------- ---------- -
SPEC/251_(Genotype_XIX)           ---------- ---------- ---------- ---------- ---------- ---------- ---------- ---------- ---------- -
Pretoriuskop/96/4_(Genotype_XX)   .......... .......... .......... .......... .......... .......... .......... .......... .......... 990
RSA/1/96_(Genotype_XXI)           ---------- ---------- ---------- ---------- ---------- ---------- ---------- ---------- ---------- -
SPEC/245_(Genotype_XXII)          ---------- ---------- ---------- ---------- ---------- ---------- ---------- ---------- ---------- -
ETH/017_(Genotype_XXIIIa)         .......... .......... .......... .......... .......... .......... .......... .......... .......... 990
ETH/AA_(Genotype_XXIIIb)          .......... .......... .......... .......... .......... .......... .......... .......... .......... 990
MOZ_10/2006_(Genotype_XXIV)       ---------- ---------- ---------- ---------- ---------- ---------- ---------- ---------- ---------- -
Conservation

                                  1,000      1,020      1,040      1,060      1,080
Benin_97/1_(Genotype_I)           ATTAAGCTGC GCTTTTGGTT TAACGAGAAC GTGAACCTTG CTATTCCCTC GGTATCCATT CCCTTCGGCG AGCGCTTTAT CACCATAAAG 1080
ASFV_Georgia_2007/1_(Genotype_II) ......T... .......... ...T...... .......... .......... A......... .......... .......... .......... 1080
Warmbaths_(Genotype_III)          .......... .......... .......... .......... .......... .........C .......... .......... .......... 1080
Warthog_(Genotype_IV)             .......... .......... .......... .......... .......... A......... .......... .......... .......... 1080
Tengani_62_(Genotype_V)           .......... .......... .......... .......... .......... .......... .......... .......... .......... 1080
MOZ/94/1_(Genotype_VI)            ---------- ---------- ---------- ---------- ---------- ---------- ---------- ---------- ---------- -
Mkuzi_1979_(Genotype_VII)         .......... .......... .......... .......... .......... .......... .......... .......... .......... 1080
Malawi_Lil-20/1_(Genotype_VIII)   .......... .......... ...T...... .......... .......... .......... .......... .......... .......... 1080
Ken06.Bus_(Genotype_IX)           .......... .......... ...T...... .......... .......... .......... .......... .......... .......... 1080
Kenya_1950_(Genotype_X)           .......... .......... ...T...... .......... .......... .......... .......... .......... .......... 1080
KAB/62_(Genotype_XI)              ---------- ---------- ---------- ---------- ---------- ---------- ---------- ---------- ---------- -
MZI/921_(Genotype_XII)            ---------- ---------- ---------- ---------- ---------- ---------- ---------- ---------- ---------- -
SUM/1411_(Genotype_XIII)          ---------- ---------- ---------- ---------- ---------- ---------- ---------- ---------- ---------- -
NYA/12_(Genotype_XIV)             ---------- ---------- ---------- ---------- ---------- ---------- ---------- ---------- ---------- -
TAN/08/Mazimbu_(Genotype_XV)      G......... .......... ...T...... .......... .......... .......... .......... .......... .......... 1080
TAN/2003/2_(Genotype_XVI)         ---------- ---------- ---------- ---------- ---------- ---------- ---------- ---------- ---------- -
ZIM/92/1_(Genotype_XVII)          ---------- ---------- ---------- ---------- ---------- ---------- ---------- ---------- ---------- -
NAM/1/95_(Genotype_XVIII)         ---------- ---------- ---------- ---------- ---------- ---------- ---------- ---------- ---------- -
SPEC/251_(Genotype_XIX)           ---------- ---------- ---------- ---------- ---------- ---------- ---------- ---------- ---------- -
Pretoriuskop/96/4_(Genotype_XX)   .......... .......... .......... .......... .......T.. A......... .......... .......... .......... 1080
RSA/1/96_(Genotype_XXI)           ---------- ---------- ---------- ---------- ---------- ---------- ---------- ---------- ---------- -
SPEC/245_(Genotype_XXII)          ---------- ---------- ---------- ---------- ---------- ---------- ---------- ---------- ---------- -
ETH/017_(Genotype_XXIIIa)         .......... .......... ...T...... .......... .......... ...G...... .......... .......... .......... 1080
ETH/AA_(Genotype_XXIIIb)          .......... .......... ...T...... .......... .......... ...G...... .......... .......... .......... 1080
MOZ_10/2006_(Genotype_XXIV)       ---------- ---------- ---------- ---------- ---------- ---------- ---------- ---------- ---------- -
Conservation
```

```
                                    1,100               1,120               1,140               1,160
Benin_97/1_(Genotype_I)             CTTGCATCGC AAAAGGATTT GGTGAATGAA TTTCCTGGAC TTTTTATACG CCAGTCGCGT TTTATACCTG GACGCCCCAG TAGACGCAAT 1170
ASFV_Georgia_2007/1_(Genotype_II)   .......... .......... .......... .......... .....G.... ......A... ......G... .......... .......... 1170
Warmbaths_(Genotype_III)            .......... .......... .......... .......... .....G.... ......A... ......G... .......... .......... 1170
Warthog_(Genotype_IV)               .......... .......... .......... .......... .....G.... ......A... ......G... .......... .......... 1170
Tengani_62_(Genotype_V)             .......... .......... .......... .......... .....G.... ......A... ......G... .......... .......... 1170
MOZ/94/1_(Genotype_VI)              ---------- ---------- ---------- ---------- ---------- ---------- ---------- ---------- ---------- -
Mkuzi_1979_(Genotype_VII)           .......... .......... .......... .......... .......... ......A... .......... .......... .......... 1170
Malawi_Lil-20/1_(Genotype_VIII)     .......... .......... .........G .......... .....G.T.. ......A... .......... .......... .......... 1170
Ken06.Bus_(Genotype_IX)             .......... .......... .......... .....C.... .......C.. ......A... .......... .G........ .........C 1170
Kenya_1950_(Genotype_X)             .......... .......... .......... .....C.... .......C.. ......A... .......... .G........ .........C 1170
KAB/62_(Genotype_XI)                ---------- ---------- ---------- ---------- ---------- ---------- ---------- ---------- ---------- -
MZI/921_(Genotype_XII)              ---------- ---------- ---------- ---------- ---------- ---------- ---------- ---------- ---------- -
SUM/1411_(Genotype_XIII)            ---------- ---------- ---------- ---------- ---------- ---------- ---------- ---------- ---------- -
NYA/12_(Genotype_XIV)               ---------- ---------- ---------- ---------- ---------- ---------- ---------- ---------- ---------- -
TAN/08/Mazimbu_(Genotype_XV)        .......... .......... .........G .......... .....G.T.. ......A... .......... .......... .......... 1170
TAN/2003/2_(Genotype_XVI)           ---------- ---------- ---------- ---------- ---------- ---------- ---------- ---------- ---------- -
ZIM/92/1_(Genotype_XVII)            ---------- ---------- ---------- ---------- ---------- ---------- ---------- ---------- ---------- -
NAM/1/95_(Genotype_XVIII)           ---------- ---------- ---------- ---------- ---------- ---------- ---------- ---------- ---------- -
SPEC/251_(Genotype_XIX)             ---------- ---------- ---------- ---------- ---------- ---------- ---------- ---------- ---------- -
Pretoriuskop/96/4_(Genotype_XX)     .......... .......... .......... .......... .....G.... ......A... ......G... .......... .......... 1170
RSA/1/96_(Genotype_XXI)             ---------- ---------- ---------- ---------- ---------- ---------- ---------- ---------- ---------- -
SPEC/245_(Genotype_XXII)            ---------- ---------- ---------- ---------- ---------- ---------- ---------- ---------- ---------- -
ETH/017_(Genotype_XXIIIa)           .......... .......... .......... .......... .......T.. ......A... .......... .G........ .........C 1170
ETH/AA_(Genotype_XXIIIb)            .......... .......... .......... .......... .......T.. ......A... .......... .G........ .........C 1170
MOZ_10/2006_(Genotype_XXIV)         ---------- ---------- ---------- ---------- ---------- ---------- ---------- ---------- ---------- -
Conservation

                                         1,180               1,200               1,220               1,240               1,260
Benin_97/1_(Genotype_I)             ATACGCTTTA AACCATGGTT TATCCCAGGA GTCATTAATG AAATCTCGCT CACGAATAAT GAACTTTACA TCAATAACCT GTTTGTAACC 1260
ASFV_Georgia_2007/1_(Genotype_II)   .......... .......... .......... .......... .......... .......... .......... .......... .......... 1260
Warmbaths_(Genotype_III)            .......... .......... .......... .......... .......... .......... ..G....... .......... .......... 1260
Warthog_(Genotype_IV)               .......... .......... .......... .......... .......... .......... ..G....... .......... .......... 1260
Tengani_62_(Genotype_V)             .......... .......... .......... .......... .......A.. .......... ..G....... .......... .......... 1260
MOZ/94/1_(Genotype_VI)              ---------- ---------- ---------- ---------- ---------- ---------- ---------- ---------- ---------- -
Mkuzi_1979_(Genotype_VII)           .......... .......... .......... .......... .......... .......... .......... .......... .......... 1260
Malawi_Lil-20/1_(Genotype_VIII)     .......... .......... .......... .......... .......... .......... ..G....... .......... .......... 1260
Ken06.Bus_(Genotype_IX)             ..C....... .......... .......... .......G.. .......... .......... ..G....... .......... .......... 1260
Kenya_1950_(Genotype_X)             ..C....... .......... .......... .......G.. .......... .......... ..G....... .......... .......... 1260
KAB/62_(Genotype_XI)                ---------- ---------- ---------- ---------- ---------- ---------- ---------- ---------- ---------- -
MZI/921_(Genotype_XII)              ---------- ---------- ---------- ---------- ---------- ---------- ---------- ---------- ---------- -
SUM/1411_(Genotype_XIII)            ---------- ---------- ---------- ---------- ---------- ---------- ---------- ---------- ---------- -
NYA/12_(Genotype_XIV)               ---------- ---------- ---------- ---------- ---------- ---------- ---------- ---------- ---------- -
TAN/08/Mazimbu_(Genotype_XV)        ..T....... .......... .......... .......... .......A.. .......... ..G....... .......... .......... 1260
TAN/2003/2_(Genotype_XVI)           ---------- ---------- ---------- ---------- ---------- ---------- ---------- ---------- ---------- -
ZIM/92/1_(Genotype_XVII)            ---------- ---------- ---------- ---------- ---------- ---------- ---------- ---------- ---------- -
NAM/1/95_(Genotype_XVIII)           ---------- ---------- ---------- ---------- ---------- ---------- ---------- ---------- ---------- -
SPEC/251_(Genotype_XIX)             ---------- ---------- ---------- ---------- ---------- ---------- ---------- ---------- ---------- -
Pretoriuskop/96/4_(Genotype_XX)     .......... .......... .......... ..T....... .......... .......... .......... .......... .......... 1260
RSA/1/96_(Genotype_XXI)             ---------- ---------- ---------- ---------- ---------- ---------- ---------- ---------- ---------- -
SPEC/245_(Genotype_XXII)            ---------- ---------- ---------- ---------- ---------- ---------- ---------- ---------- ---------- -
ETH/017_(Genotype_XXIIIa)           .......... .......... .......... .......GC. .......... .......... ..G....... .......... .......... 1260
ETH/AA_(Genotype_XXIIIb)            .......... .......... .......... .......G.. .......... .......... ..G....... .......... .......... 1260
MOZ_10/2006_(Genotype_XXIV)         ---------- ---------- ---------- ---------- ---------- ---------- ---------- ---------- ---------- -
Conservation

                                                   1,280               1,300               1,320               1,340
Benin_97/1_(Genotype_I)             CCTGAAATAC ACAACCTTTT TGTAAAACGC GTTCGATTTT CCCTGATACG TGTCCATAAA ACGCAGGTGA CCCACACCAA CAATAACCAC 1350
ASFV_Georgia_2007/1_(Genotype_II)   .......... .......... .......... .....C.... .G........ .......... .......... .......... .......... 1350
Warmbaths_(Genotype_III)            .......... .......... .......... .......... .T........ .......... .......... .......... .......... 1350
Warthog_(Genotype_IV)               ........T. .......... .......... .......... .T........ .......... .......... ....T..... .......... 1350
Tengani_62_(Genotype_V)             .......... .......... .......... .......... .T........ .......... .......... .......... .......... 1350
MOZ/94/1_(Genotype_VI)              ---------- ---------- ---------- ---------- ---------- ---------- ---------- ---------- ---------- -
Mkuzi_1979_(Genotype_VII)           .......... .......... .......... .......... .......... C......... .......... .......... .......... 1350
Malawi_Lil-20/1_(Genotype_VIII)     .......... .......... .......... .....G.... .......... .......... .......... .......G.. ......T..T 1350
Ken06.Bus_(Genotype_IX)             .......... ....T..... .......... .......... .......... .......... .......... .......G.. .........T 1350
Kenya_1950_(Genotype_X)             .......... .......... C......... .......... .......... .......... .......... .......G.. T........T 1350
KAB/62_(Genotype_XI)                ---------- ---------- ---------- ---------- ---------- ---------- ---------- ---------- ---------- -
MZI/921_(Genotype_XII)              ---------- ---------- ---------- ---------- ---------- ---------- ---------- ---------- ---------- -
SUM/1411_(Genotype_XIII)            ---------- ---------- ---------- ---------- ---------- ---------- ---------- ---------- ---------- -
NYA/12_(Genotype_XIV)               ---------- ---------- ---------- ---------- ---------- ---------- ---------- ---------- ---------- -
TAN/08/Mazimbu_(Genotype_XV)        .......... .......... .......... .....G.... .......... .......... .......... .......G.. .........T 1350
TAN/2003/2_(Genotype_XVI)           ---------- ---------- ---------- ---------- ---------- ---------- ---------- ---------- ---------- -
ZIM/92/1_(Genotype_XVII)            ---------- ---------- ---------- ---------- ---------- ---------- ---------- ---------- ---------- -
NAM/1/95_(Genotype_XVIII)           ---------- ---------- ---------- ---------- ---------- ---------- ---------- ---------- ---------- -
SPEC/251_(Genotype_XIX)             ---------- ---------- ---------- ---------- ---------- ---------- ---------- ---------- ---------- -
Pretoriuskop/96/4_(Genotype_XX)     .......... .......... .......... .......... .T........ .......... .......... .......... .......... 1350
RSA/1/96_(Genotype_XXI)             ---------- ---------- ---------- ---------- ---------- ---------- ---------- ---------- ---------- -
SPEC/245_(Genotype_XXII)            ---------- ---------- ---------- ---------- ---------- ---------- ---------- ---------- ---------- -
ETH/017_(Genotype_XXIIIa)           .......... .......... .......... .......... .......... C......... .......... .......G.. .........T 1350
ETH/AA_(Genotype_XXIIIb)            .......... .......... .......... ..A....... .......... C......... .......... .......G.. .........T 1350
MOZ_10/2006_(Genotype_XXIV)         ---------- ---------- ---------- ---------- ---------- ---------- ---------- ---------- ---------- -
Conservation
```

Sequence (positions 1,351–1,440; ticks at 1,360 / 1,380 / 1,400 / 1,420 / 1,440)	Alignment	End
Benin_97/1_(Genotype_I)	CACGATGAAA AACTAATGTC TGCTCTTAAA TGGCCCATTG AATATATGTT TATAGGATTA AAACCTACCT GGAACATCTC CGATCAAAAT	1440
ASFV_Georgia_2007/1_(Genotype_II)	....	1440
Warmbaths_(Genotype_III)	....	1440
Warthog_(Genotype_IV)	....	1440
Tengani_62_(Genotype_V)	....	1440
MOZ/94/1_(Genotype_VI)	---------- ---------- ---------- ---------- ---------- ---------- ---------- ---------- ----------	–
Mkuzi_1979_(Genotype_VII)	....	1440
Malawi_Lil-20/1_(Genotype_VIII)	....T.	1440
Ken06.Bus_(Genotype_IX)	....C. ....C.... ....T..T.. ...C......	1440
Kenya_1950_(Genotype_X)	....C. ....C.... ....T..T.. ...C......	1440
KAB/62_(Genotype_XI)	---------- ---------- ---------- ---------- ---------- ---------- ---------- ---------- ----------	–
MZI/921_(Genotype_XII)	---------- ---------- ---------- ---------- ---------- ---------- ---------- ---------- ----------	–
SUM/1411_(Genotype_XIII)	---------- ---------- ---------- ---------- ---------- ---------- ---------- ---------- ----------	–
NYA/12_(Genotype_XIV)	---------- ---------- ---------- ---------- ---------- ---------- ---------- ---------- ----------	–
TAN/08/Mazimbu_(Genotype_XV)	....G... ....T.	1440
TAN/2003/2_(Genotype_XVI)	---------- ---------- ---------- ---------- ---------- ---------- ---------- ---------- ----------	–
ZIM/92/1_(Genotype_XVII)	---------- ---------- ---------- ---------- ---------- ---------- ---------- ---------- ----------	–
NAM/1/95_(Genotype_XVIII)	---------- ---------- ---------- ---------- ---------- ---------- ---------- ---------- ----------	–
SPEC/251_(Genotype_XIX)	---------- ---------- ---------- ---------- ---------- ---------- ---------- ---------- ----------	–
Pretoriuskop/96/4_(Genotype_XX)	....	1440
RSA/1/96_(Genotype_XXI)	---------- ---------- ---------- ---------- ---------- ---------- ---------- ---------- ----------	–
SPEC/245_(Genotype_XXII)	---------- ---------- ---------- ---------- ---------- ---------- ---------- ---------- ----------	–
ETH/017_(Genotype_XXIIIa)	....C. ....C.... ....T..T.. ...C......	1440
ETH/AA_(Genotype_XXIIIb)	....C. ....C.... ....T..T.. ...C......	1440
MOZ_10/2006_(Genotype_XXIV)	---------- ---------- ---------- ---------- ---------- ---------- ---------- ---------- ----------	–
Conservation		

Sequence (positions 1,441–1,530; ticks at 1,460 / 1,480 / 1,500 / 1,520)	Alignment	End
Benin_97/1_(Genotype_I)	CCTCATCAAC ACCGAGATTG GCACAAGTTC GGACATGTTG TTAACGCCAT TATGCAGCCT ACTCACCACG CAGAGATAAG CTTTCAGGAT	1530
ASFV_Georgia_2007/1_(Genotype_II)	....C	1530
Warmbaths_(Genotype_III)	....	1530
Warthog_(Genotype_IV)	....	1530
Tengani_62_(Genotype_V)	....	1530
MOZ/94/1_(Genotype_VI)	---------- ---------- ---------- ---------- ---------- -.........	39
Mkuzi_1979_(Genotype_VII)	........G.	1530
Malawi_Lil-20/1_(Genotype_VIII)	....C.... ....A.....	1530
Ken06.Bus_(Genotype_IX)	....C....... .....G....	1530
Kenya_1950_(Genotype_X)	....C....... .....G....	1530
KAB/62_(Genotype_XI)	---------- ---------- ---------- ---------- ---------- -......... ....A.....	39
MZI/921_(Genotype_XII)	---------- ---------- ---------- ---------- ---------- -......... ....A.....	39
SUM/1411_(Genotype_XIII)	---------- ---------- ---------- ---------- ---------- -......... ....A.....	39
NYA/12_(Genotype_XIV)	---------- ---------- ---------- ---------- ---------- -......... ....A.....	39
TAN/08/Mazimbu_(Genotype_XV)	....A..... C	1530
TAN/2003/2_(Genotype_XVI)	---------- ---------- ---------- ---------- ---------- -.........	39
ZIM/92/1_(Genotype_XVII)	---------- ---------- ---------- ---------- ---------- ----......	36
NAM/1/95_(Genotype_XVIII)	---------- ---------- ---------- ---------- ---------- ----......	36
SPEC/251_(Genotype_XIX)	---------- ---------- ---------- ---------- ---------- ----......	36
Pretoriuskop/96/4_(Genotype_XX)	....	1530
RSA/1/96_(Genotype_XXI)	---------- ---------- ---------- ---------- ---------- ----....T.	36
SPEC/245_(Genotype_XXII)	---------- ---------- ---------- ---------- ---------- ----......	36
ETH/017_(Genotype_XXIIIa)	....G....... ....C....... .....G....	1530
ETH/AA_(Genotype_XXIIIb)	....G....... ....C....... .....G....	1530
MOZ_10/2006_(Genotype_XXIV)	---------- ---------- ---------- ---------- ---------- ----......	36
Conservation		

Sequence (positions 1,531–1,620; ticks at 1,540 / 1,560 / 1,580 / 1,600 / 1,620)	Alignment	End
Benin_97/1_(Genotype_I)	AGAGATACAG CTCTTCCAGA CGCATGTTCA TCTATATCGG ATATTAGCCC CGTTACGTAT CCGATCACAT TACCTATTAT TAAAAACATT	1620
ASFV_Georgia_2007/1_(Genotype_II)	....T.	1620
Warmbaths_(Genotype_III)	....T. ....G.	1620
Warthog_(Genotype_IV)	....T. .... .A........ ....G.	1620
Tengani_62_(Genotype_V)	....T. ....C...... .....T....	1620
MOZ/94/1_(Genotype_VI)	....T. ....C......	129
Mkuzi_1979_(Genotype_VII)	....	1620
Malawi_Lil-20/1_(Genotype_VIII)	....T. ......A... ......T...	1620
Ken06.Bus_(Genotype_IX)	.... T......... ..C.....T. ......C... .A....T...	1620
Kenya_1950_(Genotype_X)	.... T......... ..C.....A. .....TC... .A....T... ....G.	1620
KAB/62_(Genotype_XI)	....T. ......A... ......T...	129
MZI/921_(Genotype_XII)	....G...... ....T. ......A... ......T...	129
SUM/1411_(Genotype_XIII)	....T. ......A... ......T...	129
NYA/12_(Genotype_XIV)	....T. ......A...	129
TAN/08/Mazimbu_(Genotype_XV)	....T. ......AT.. ......T...	1620
TAN/2003/2_(Genotype_XVI)	....T. ......A... ......T...	129
ZIM/92/1_(Genotype_XVII)	.... .A....T...	126
NAM/1/95_(Genotype_XVIII)	....T.	126
SPEC/251_(Genotype_XIX)	....T. ....G.	126
Pretoriuskop/96/4_(Genotype_XX)	....T. ....G.	1620
RSA/1/96_(Genotype_XXI)	....T. .... .A........	126
SPEC/245_(Genotype_XXII)	....T.	126
ETH/017_(Genotype_XXIIIa)	.... T......... ..C.....T. ......C... .A....T... ..A.......	1620
ETH/AA_(Genotype_XXIIIb)	.... T......... ..C.....T. ......C... .A....T... .....T....	1620
MOZ_10/2006_(Genotype_XXIV)	....T. .... .A........	126
Conservation		

```
                                     1,640                1,660                1,680                1,700
Benin_97/1_(Genotype_I)              TCCGTAACTG CTCATGGTAT CAATCTTATC GATAAGTTTC CATCAAAGTT CTGCAGCTCT TACATACCCT TCCACTACGG AGGCAATGCA 1710
ASFV_Georgia_2007/1_(Genotype_II)    .......... .......... .......... .....A.... .......... .......... .......... .......... .........G 1710
Warmbaths_(Genotype_III)             .......... .......... .......... .....A.... .......... .......... .......... .......... .........G 1710
Warthog_(Genotype_IV)                .......... .......... .......... .....A.... .......... .......... .......... .......... .........G 1710
Tengani_62_(Genotype_V)              .......... .......... .......... .....A.... .......... .......... ........T. .......... .........G 1710
MOZ/94/1_(Genotype_VI)               ..T....... .......... .......... .....A.... .......... .......... ........T. .......... .........G 219
Mkuzi_1979_(Genotype_VII)            .......... .......... .......... .......... .......... .......... .......... .......... .....G.... 1710
Malawi_Lil-20/1_(Genotype_VIII)      .......... .......... .......... .......... .......... .......... .......... .......T.. .......T.G 1710
Ken06.Bus_(Genotype_IX)              .....T.... ....C..... .......... .....A.... .......... .......... .......... .......... .......T.G 1710
Kenya_1950_(Genotype_X)              .....C.... .......... .......... .....A.... .......... .......... .......... .T........ .......T.G 1710
KAB/62_(Genotype_XI)                 .......... .......... .......... .......... .......... .......... .......... .......... .......T.G 219
MZI/921_(Genotype_XII)               .......... .......... .......... .......... .......... .......... .......... .......... .......T.G 219
SUM/1411_(Genotype_XIII)             .......... .......... .......... .......... .......... .......... .......... .......... .......T.G 219
NYA/12_(Genotype_XIV)                .......... .......... .......... .......... .......... .......... .......... .......... ...T...T.G 219
TAN/08/Mazimbu_(Genotype_XV)         ........A. .......G.. .......... .......... .......... .......... .......... .......... .......T.G 1710
TAN/2003/2_(Genotype_XVI)            .......... .......... .......... .......... .......... .......... .......... .......... .......T.G 219
ZIM/92/1_(Genotype_XVII)             .......... .......... .......... .......... .......... .......... .......... .......... .........G 216
NAM/1/95_(Genotype_XVIII)            .......... .......... .......... .......... .......... .......... .......... .......... .........G 216
SPEC/251_(Genotype_XIX)              .......... .......... .......... .....A.... .......... .......... .......... .......... .........G 216
Pretoriuskop/96/4_(Genotype_XX)      .......... .......... .......... .....A.... .......... .......... .......... .......... .........G 1710
RSA/1/96_(Genotype_XXI)              .......... .......... .......... .....A.... .......... .......... .......... .......... .........G 216
SPEC/245_(Genotype_XXII)             .......... .......... .......... .....A.... .......... .......... .......... .......... .....G...G 216
ETH/017_(Genotype_XXIIIa)            .....T.... .......C.. .......... .....A.... .......... .......... .......... .......... .......T.G 1710
ETH/AA_(Genotype_XXIIIb)             .....T.... .......C.. .......... .....A.... .......... .......... .......... .......... .......T.G 1710
MOZ_10/2006_(Genotype_XXIV)          .......... .C........ .......... .....A.... .......... .......... .......... .......... .........G 216
Conservation

                                          1,720                1,740                1,760                1,780               1,800
Benin_97/1_(Genotype_I)              ATTAAAACCC CCGATGATCC GGGTGCGATG ATGATTACCT TTGCTTTGAA GCCACGGGAG GAATACCAAC CCAGTGGTCA TATTAACGTA 1800
ASFV_Georgia_2007/1_(Genotype_II)    .......... .......... .......... .......... .......... .......... .......... .......... .......... 1800
Warmbaths_(Genotype_III)             .......... ....C..... .......... .......... .......... .......... .......... .......... .......... 1800
Warthog_(Genotype_IV)                .......... ....C..... .......... .......... .......... .......... .......... .......... ......T... 1800
Tengani_62_(Genotype_V)              .......... .......... .......... .......... ....C..... .......... .......... .......... .......... 1800
MOZ/94/1_(Genotype_VI)               .......... ....C..... .......... .......... ....C..... .......... .......... .......... .......... 309
Mkuzi_1979_(Genotype_VII)            .......... .......... .......... .......... .......... .......... .......... .......... .......... 1800
Malawi_Lil-20/1_(Genotype_VIII)      .......... .T........ ...C...... .......... .C........ .......... .......... .......... .......... 1800
Ken06.Bus_(Genotype_IX)              .......... ....C..... ...C...... .......... .......... A......... .......... ....C..... .......... 1800
Kenya_1950_(Genotype_X)              .......... ....C..... ...C...... .......... .......... A......... .......... .......... .......... 1800
KAB/62_(Genotype_XI)                 .......... ....C..... ...C...... .......... .C........ .......... .......... .......... .......... 309
MZI/921_(Genotype_XII)               .......... ....C..... ...C...... .......... .C........ .......... .......... .......... .......... 309
SUM/1411_(Genotype_XIII)             .......... ....C..... ...C...... .......... .C........ .......... .......... .......... .......... 309
NYA/12_(Genotype_XIV)                ........T. .......... ...C...... .......... .......... .......... .......... .......... .......... 309
TAN/08/Mazimbu_(Genotype_XV)         .......... .T..C..... ...C...... .......... .C........ .......... .......... .......... .......... 1800
TAN/2003/2_(Genotype_XVI)            ........T. .T..C..... ...C...... .......... .C........ .......... .......... .......... .......... 309
ZIM/92/1_(Genotype_XVII)             .......... .......... .......... .......... .......... .......... .......... .......... .......... 306
NAM/1/95_(Genotype_XVIII)            .......... .......... .......... .......... .C........ .......... .......... .......... .......... 306
SPEC/251_(Genotype_XIX)              .......... ....C..... .......... .......... .......... .......... .......... .......... ......T... 306
Pretoriuskop/96/4_(Genotype_XX)      .......... ....C..... .......... .......... .......... .......... .......... .......... .......... 1800
RSA/1/96_(Genotype_XXI)              .......... ....C..... .......... .......... .......... .......... .......... .......... .......... 306
SPEC/245_(Genotype_XXII)             .......... .T..C..... T......... .......... .......... .......... .......... .......... ......T... 306
ETH/017_(Genotype_XXIIIa)            .......... ....C..... ...C...... .......... .......... .......... .......... .......C.. .......... 1800
ETH/AA_(Genotype_XXIIIb)             .......... ....C..... ...C...... .......... .......... .......... .......... .......C.. .......... 1800
MOZ_10/2006_(Genotype_XXIV)          .......... ....C..... .......... .......... .......... .......... .......... .......... .......... 306
Conservation

                                                     1,820                1,840                1,860                1,880
Benin_97/1_(Genotype_I)              TCCAGAGCAA GAGAATTTTA TATTAGTTGG GACACGGATT ACGTGGGGTC TATCACTACG GCTGATCTTG TGGTATCGGC ATCTGCTATT 1890
ASFV_Georgia_2007/1_(Genotype_II)    .......... .......... .......... .......... .......... .......... .......... .......... .......... 1890
Warmbaths_(Genotype_III)             .......... .......... .......... ..T....... .......... ......C... .......... .......... .......... 1890
Warthog_(Genotype_IV)                ..T....... .......... .......... ..T....... .......... .......... .......... .......... .......... 1890
Tengani_62_(Genotype_V)              ..T....... .......... .......... ..T....... .......... .......... .......... .......A.. .......... 1890
MOZ/94/1_(Genotype_VI)               ..T....... .......... .......... ..T....... .......... .......... .......... .......... .......... 399
Mkuzi_1979_(Genotype_VII)            .......... .......... .......... .......... .......... .......... .......... .......... .......... 1890
Malawi_Lil-20/1_(Genotype_VIII)      .......... .......... .......... .......... .T.....A.. ...T..C... .......... .......A.. .......... 1890
Ken06.Bus_(Genotype_IX)              .......... .......... ......C... .....A.... .T........ ......C... .......... .......... ...C...... 1890
Kenya_1950_(Genotype_X)              .......... ....G..... ......C... ..T..A.... .T........ ......C... ..C....... .......... ...C...... 1890
KAB/62_(Genotype_XI)                 .......... .......... .......... .......... .T........ ...T..C... .......... .......A.. .......... 399
MZI/921_(Genotype_XII)               .......... .......... .......... .......... .T........ ...T..C... .......... .......... .......... 399
SUM/1411_(Genotype_XIII)             .......... .......... .......... .......... .T........ ......C... ..G....... ...C..GC.G .......... 399
NYA/12_(Genotype_XIV)                .......... .......... .......... .......... .T........ ......C... .......... .......... ......C... 399
TAN/08/Mazimbu_(Genotype_XV)         .......... .......... ......C... .......... .T.....A.. ......C... .......... .......... .......... 1890
TAN/2003/2_(Genotype_XVI)            .......... .......... .......... .......... .T........ ......C... .......... .......... .......... 399
ZIM/92/1_(Genotype_XVII)             .......... .......... .......... .......... .......... .......... .......... .......... .......... 396
NAM/1/95_(Genotype_XVIII)            .......... .......... .......... .......... .......... ...T..C... .......... .......A.. .......... 396
SPEC/251_(Genotype_XIX)              ..T....... .......... .......... ..T....... .......... ......C... .......... .......... .......... 396
Pretoriuskop/96/4_(Genotype_XX)      ..T....... .......... .......... ..T....... .......... .......... .......... .......... .......... 1890
RSA/1/96_(Genotype_XXI)              ..T....... .......... .......... ..T....... .......... .......... .......... .......... .......... 396
SPEC/245_(Genotype_XXII)             ..T....... .......... .......... ..T....... .......... ......C... .......... .......... .......... 396
ETH/017_(Genotype_XXIIIa)            ..T....... .......... ......C... ........A. .T........ ......C... .......... .......... ...C...... 1890
ETH/AA_(Genotype_XXIIIb)             ..T....... .......... .......... ........A. .T........ ......C... .......... .......... ...C...... 1890
MOZ_10/2006_(Genotype_XXIV)          .......... .......... .......... ..T....... .......... C......... .......... .......... .......... 396
Conservation
```

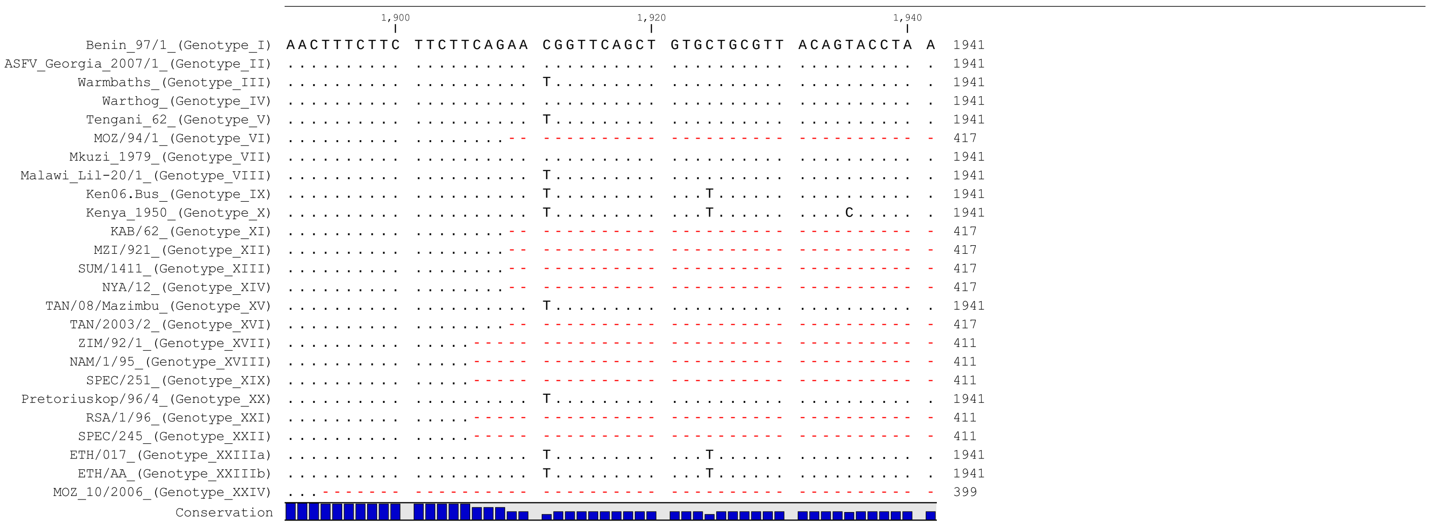

1,900
1,920
1,940
Benin_97/1_(Genotype_I) AACTTTCTTC TTCTTCAGAA CGGTTCAGCT GTGCTGCGTT ACAGTACCTA A 1941
ASFV_Georgia_2007/1_(Genotype_II) 1941
Warmbaths_(Genotype_III) .... T......... 1941
Warthog_(Genotype_IV) 1941
Tengani_62_(Genotype_V) .... T......... 1941
MOZ/94/1_(Genotype_VI) ....-- ---------- ---------- ---------- - 417
Mkuzi_1979_(Genotype_VII) 1941
Malawi_Lil-20/1_(Genotype_VIII) .... T......... 1941
Ken06.Bus_(Genotype_IX) .... T......... ...T...... 1941
Kenya_1950_(Genotype_X) .... T......... ...T...... ....C..... . 1941
KAB/62_(Genotype_XI) ....-- ---------- ---------- ---------- - 417
MZI/921_(Genotype_XII) ....-- ---------- ---------- ---------- - 417
SUM/1411_(Genotype_XIII) ....-- ---------- ---------- ---------- - 417
NYA/12_(Genotype_XIV) ....-- ---------- ---------- ---------- - 417
TAN/08/Mazimbu_(Genotype_XV) .... T......... 1941
TAN/2003/2_(Genotype_XVI) ....-- ---------- ---------- ---------- - 417
ZIM/92/1_(Genotype_XVII) ....----- ---------- ---------- ---------- - 411
NAM/1/95_(Genotype_XVIII) ....----- ---------- ---------- ---------- - 411
SPEC/251_(Genotype_XIX) ....----- ---------- ---------- ---------- - 411
Pretoriuskop/96/4_(Genotype_XX) .... T......... 1941
RSA/1/96_(Genotype_XXI) ....----- ---------- ---------- ---------- - 411
SPEC/245_(Genotype_XXII) ....----- ---------- ---------- ---------- - 411
ETH/017_(Genotype_XXIIIa) .... T......... ...T...... 1941
ETH/AA_(Genotype_XXIIIb) .... T......... ...T...... 1941
MOZ_10/2006_(Genotype_XXIV) ...------- ---------- ---------- ---------- ---------- - 399
Conservation
