# Supplementary material for: A Re-Evaluation of African Swine Fever Genotypes Based on p72 Sequences Reveals the Existence of Only Six Distinct p72 Groups
Source: Viruses. 2023 Nov 11;15(11):2246. doi: 10.3390/v15112246 (PMC10675559; doi:10.3390/v15112246)
Supplement: Supplementary file 1 [file viruses-15-02246-s001.zip › Figure S2 Alignment of historical genotypes (protein).pdf]

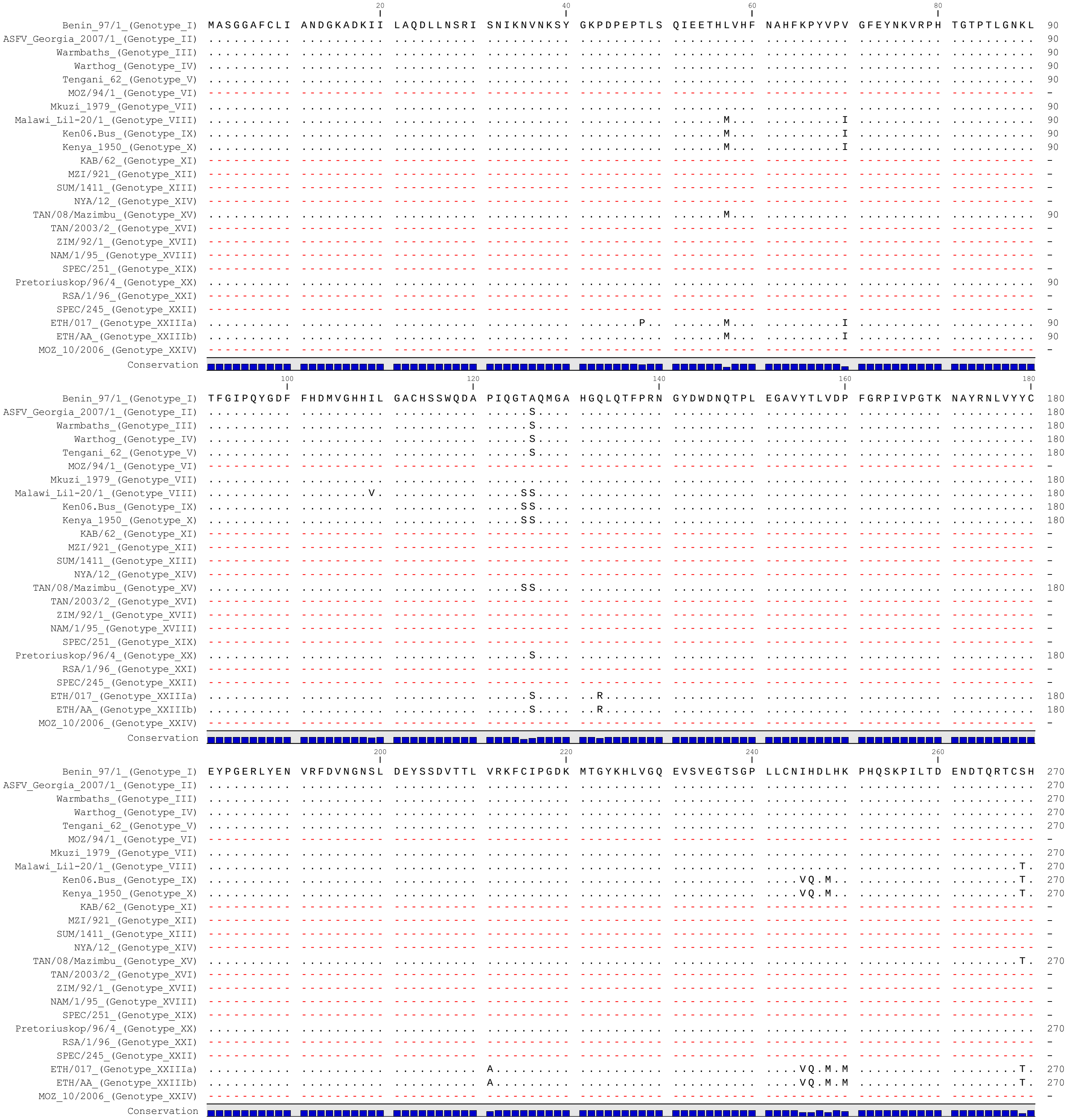
20 40 60 80
Benin_97/1_(Genotype_I) MASGGAFCLI ANDGKADKII LAQDLLNSRI SNIKNVNKSY GKPDPEPTLS QIEETHLVHF NAHFKPYVPV GFEYNKVRPH TGTPTLGNKL 90
ASFV_Georgia_2007/1_(Genotype_II) 90
Warmbaths_(Genotype_III) 90
Warthog_(Genotype_IV) 90
Tengani_62_(Genotype_V) 90
MOZ/94/1_(Genotype_VI) ---------- ---------- ---------- ---------- ---------- ---------- ---------- ---------- ---------- -
Mkuzi_1979_(Genotype_VII) 90
Malawi_Lil-20/1_(Genotype_VIII) ....M... ....I 90
Ken06.Bus_(Genotype_IX) ....M... ....I 90
Kenya_1950_(Genotype_X) ....M... ....I 90
KAB/62_(Genotype_XI) ---------- ---------- ---------- ---------- ---------- ---------- ---------- ---------- ---------- -
MZI/921_(Genotype_XII) ---------- ---------- ---------- ---------- ---------- ---------- ---------- ---------- ---------- -
SUM/1411_(Genotype_XIII) ---------- ---------- ---------- ---------- ---------- ---------- ---------- ---------- ---------- -
NYA/12_(Genotype_XIV) ---------- ---------- ---------- ---------- ---------- ---------- ---------- ---------- ---------- -
TAN/08/Mazimbu_(Genotype_XV) ....M... 90
TAN/2003/2_(Genotype_XVI) ---------- ---------- ---------- ---------- ---------- ---------- ---------- ---------- ---------- -
ZIM/92/1_(Genotype_XVII) ---------- ---------- ---------- ---------- ---------- ---------- ---------- ---------- ---------- -
NAM/1/95_(Genotype_XVIII) ---------- ---------- ---------- ---------- ---------- ---------- ---------- ---------- ---------- -
SPEC/251_(Genotype_XIX) ---------- ---------- ---------- ---------- ---------- ---------- ---------- ---------- ---------- -
Pretoriuskop/96/4_(Genotype_XX) 90
RSA/1/96_(Genotype_XXI) ---------- ---------- ---------- ---------- ---------- ---------- ---------- ---------- ---------- -
SPEC/245_(Genotype_XXII) ---------- ---------- ---------- ---------- ---------- ---------- ---------- ---------- ---------- -
ETH/017_(Genotype_XXIIIa) ....P.. ......M... ....I 90
ETH/AA_(Genotype_XXIIIb) ....M... ....I 90
MOZ_10/2006_(Genotype_XXIV) ---------- ---------- ---------- ---------- ---------- ---------- ---------- ---------- ---------- -
Conservation
100 120 140 160 180
Benin_97/1_(Genotype_I) TFGIPQYGDF FHDMVGHHIL GACHSSWQDA PIQGTAQMGA HGQLQTFPRN GYDWDNQTPL EGAVYTLVDP FGRPIVPGTK NAYRNLVYYC 180
ASFV_Georgia_2007/1_(Genotype_II) ....S.... 180
Warmbaths_(Genotype_III) ....S.... 180
Warthog_(Genotype_IV) ....S.... 180
Tengani_62_(Genotype_V) ....S.... 180
MOZ/94/1_(Genotype_VI) ---------- ---------- ---------- ---------- ---------- ---------- ---------- ---------- ---------- -
Mkuzi_1979_(Genotype_VII) 180
Malawi_Lil-20/1_(Genotype_VIII) ....V. ....SS.... 180
Ken06.Bus_(Genotype_IX) ....SS.... 180
Kenya_1950_(Genotype_X) ....SS.... 180
KAB/62_(Genotype_XI) ---------- ---------- ---------- ---------- ---------- ---------- ---------- ---------- ---------- -
MZI/921_(Genotype_XII) ---------- ---------- ---------- ---------- ---------- ---------- ---------- ---------- ---------- -
SUM/1411_(Genotype_XIII) ---------- ---------- ---------- ---------- ---------- ---------- ---------- ---------- ---------- -
NYA/12_(Genotype_XIV) ---------- ---------- ---------- ---------- ---------- ---------- ---------- ---------- ---------- -
TAN/08/Mazimbu_(Genotype_XV) ....SS.... 180
TAN/2003/2_(Genotype_XVI) ---------- ---------- ---------- ---------- ---------- ---------- ---------- ---------- ---------- -
ZIM/92/1_(Genotype_XVII) ---------- ---------- ---------- ---------- ---------- ---------- ---------- ---------- ---------- -
NAM/1/95_(Genotype_XVIII) ---------- ---------- ---------- ---------- ---------- ---------- ---------- ---------- ---------- -
SPEC/251_(Genotype_XIX) ---------- ---------- ---------- ---------- ---------- ---------- ---------- ---------- ---------- -
Pretoriuskop/96/4_(Genotype_XX) ....S.... 180
RSA/1/96_(Genotype_XXI) ---------- ---------- ---------- ---------- ---------- ---------- ---------- ---------- ---------- -
SPEC/245_(Genotype_XXII) ---------- ---------- ---------- ---------- ---------- ---------- ---------- ---------- ---------- -
ETH/017_(Genotype_XXIIIa) ....S.... ..R....... 180
ETH/AA_(Genotype_XXIIIb) ....S.... ..R....... 180
MOZ_10/2006_(Genotype_XXIV) ---------- ---------- ---------- ---------- ---------- ---------- ---------- ---------- ---------- -
Conservation
200 220 240 260
Benin_97/1_(Genotype_I) EYPGERLYEN VRFDVNGNSL DEYSSDVTTL VRKFCIPGDK MTGYKHLVGQ EVSVEGTSGP LLCNIHDLHK PHQSKPILTD ENDTQRTCSH 270
ASFV_Georgia_2007/1_(Genotype_II) 270
Warmbaths_(Genotype_III) 270
Warthog_(Genotype_IV) 270
Tengani_62_(Genotype_V) 270
MOZ/94/1_(Genotype_VI) ---------- ---------- ---------- ---------- ---------- ---------- ---------- ---------- ---------- -
Mkuzi_1979_(Genotype_VII) 270
Malawi_Lil-20/1_(Genotype_VIII) ....T. 270
Ken06.Bus_(Genotype_IX) ....VQ.M.. ....T. 270
Kenya_1950_(Genotype_X) ....VQ.M.. ....T. 270
KAB/62_(Genotype_XI) ---------- ---------- ---------- ---------- ---------- ---------- ---------- ---------- ---------- -
MZI/921_(Genotype_XII) ---------- ---------- ---------- ---------- ---------- ---------- ---------- ---------- ---------- -
SUM/1411_(Genotype_XIII) ---------- ---------- ---------- ---------- ---------- ---------- ---------- ---------- ---------- -
NYA/12_(Genotype_XIV) ---------- ---------- ---------- ---------- ---------- ---------- ---------- ---------- ---------- -
TAN/08/Mazimbu_(Genotype_XV) ....T. 270
TAN/2003/2_(Genotype_XVI) ---------- ---------- ---------- ---------- ---------- ---------- ---------- ---------- ---------- -
ZIM/92/1_(Genotype_XVII) ---------- ---------- ---------- ---------- ---------- ---------- ---------- ---------- ---------- -
NAM/1/95_(Genotype_XVIII) ---------- ---------- ---------- ---------- ---------- ---------- ---------- ---------- ---------- -
SPEC/251_(Genotype_XIX) ---------- ---------- ---------- ---------- ---------- ---------- ---------- ---------- ---------- -
Pretoriuskop/96/4_(Genotype_XX) 270
RSA/1/96_(Genotype_XXI) ---------- ---------- ---------- ---------- ---------- ---------- ---------- ---------- ---------- -
SPEC/245_(Genotype_XXII) ---------- ---------- ---------- ---------- ---------- ---------- ---------- ---------- ---------- -
ETH/017_(Genotype_XXIIIa) .... A......... ....VQ.M.M ....T. 270
ETH/AA_(Genotype_XXIIIb) .... A......... ....VQ.M.M ....T. 270
MOZ_10/2006_(Genotype_XXIV) ---------- ---------- ---------- ---------- ---------- ---------- ---------- ---------- ---------- -
Conservation

```
                                      280        300        320        340        360
Benin_97/1_(Genotype_I)               TNPKFLSQHF PENSHNIQTA GKQDITPITD ATYLDIRRNV HYSCNGPQTP KYYQPPLALW IKLRFWFNEN VNLAIPSVSI PFGERFITIK 360
ASFV_Georgia_2007/1_(Genotype_II)     .......... .......... .......... .......... .......... .......... .......... .......... .......... 360
Warmbaths_(Genotype_III)              .......... .......... .......... .......... .......... .......... .......... .......... .......... 360
Warthog_(Genotype_IV)                 .......... .......... .......... .......... .......... .......... .......... .......... .......... 360
Tengani_62_(Genotype_V)               .......... .......... .......... .......... .......... .......... .......... .......... .......... 360
MOZ/94/1_(Genotype_VI)                ---------- ---------- ---------- ---------- ---------- ---------- ---------- ---------- ---------- –
Mkuzi_1979_(Genotype_VII)             .......... .......... .......... .......... .......... .......... .......... .......... .......... 360
Malawi_Lil-20/1_(Genotype_VIII)       .......... .......... .......... T......... Q......... .......... .......... .......... .......... 360
Ken06.Bus_(Genotype_IX)               .......... .......... .......... .......... .......... .......... .......... .......... .......... 360
Kenya_1950_(Genotype_X)               .......... .......... .......... .......... .......... .......... .......... .......... .......... 360
KAB/62_(Genotype_XI)                  ---------- ---------- ---------- ---------- ---------- ---------- ---------- ---------- ---------- –
MZI/921_(Genotype_XII)                ---------- ---------- ---------- ---------- ---------- ---------- ---------- ---------- ---------- –
SUM/1411_(Genotype_XIII)              ---------- ---------- ---------- ---------- ---------- ---------- ---------- ---------- ---------- –
NYA/12_(Genotype_XIV)                 ---------- ---------- ---------- ---------- ---------- ---------- ---------- ---------- ---------- –
TAN/08/Mazimbu_(Genotype_XV)          .......... .......... .......... T......... Q......... .......... V......... .......... .......... 360
TAN/2003/2_(Genotype_XVI)             ---------- ---------- ---------- ---------- ---------- ---------- ---------- ---------- ---------- –
ZIM/92/1_(Genotype_XVII)              ---------- ---------- ---------- ---------- ---------- ---------- ---------- ---------- ---------- –
NAM/1/95_(Genotype_XVIII)             ---------- ---------- ---------- ---------- ---------- ---------- ---------- ---------- ---------- –
SPEC/251_(Genotype_XIX)               ---------- ---------- ---------- ---------- ---------- ---------- ---------- ---------- ---------- –
Pretoriuskop/96/4_(Genotype_XX)       .......... .......... .......... .......... .......... .......... .......... .......... .......... 360
RSA/1/96_(Genotype_XXI)               ---------- ---------- ---------- ---------- ---------- ---------- ---------- ---------- ---------- –
SPEC/245_(Genotype_XXII)              ---------- ---------- ---------- ---------- ---------- ---------- ---------- ---------- ---------- –
ETH/017_(Genotype_XXIIIa)             .......... .......... .......... .......... .......... .......... .......... .......... .......... 360
ETH/AA_(Genotype_XXIIIb)              .......... .......... .......... .......... .......... .......... .......... .......... .......... 360
MOZ_10/2006_(Genotype_XXIV)           ---------- ---------- ---------- ---------- ---------- ---------- ---------- ---------- ---------- –
Conservation

                                                          380                   400                   420                   440
Benin_97/1_(Genotype_I)               LASQKDLVNE FPGLFIRQSR FIPGRPSRRN IRFKPWFIPG VINEISLTNN ELYINNLFVT PEIHNLFVKR VRFSLIRVHK TQVTHTNNNH 450
ASFV_Georgia_2007/1_(Genotype_II)     .......... .....V.... ..A....... .......... .......... .......... .......... .......... .......... 450
Warmbaths_(Genotype_III)              .......... .....V.... ..A....... .......... .......... .......... .......... .......... .......... 450
Warthog_(Genotype_IV)                 .......... .....V.... ..A....... .......... .......... .......... .......... .......... .......... 450
Tengani_62_(Genotype_V)               .......... .....V.... ..A....... .......... .......... .......... .......... .......... .......... 450
MOZ/94/1_(Genotype_VI)                ---------- ---------- ---------- ---------- ---------- ---------- ---------- ---------- ---------- –
Mkuzi_1979_(Genotype_VII)             .......... .......... .......... .......... .......... .......... .......... .......... .......... 450
Malawi_Lil-20/1_(Genotype_VIII)       .......... .....V.... .......... .......... .......... .......... .......... .......... .......... 450
Ken06.Bus_(Genotype_IX)               .......... .......... .......... .......... ..S....... .......... .......... .......... .......... 450
Kenya_1950_(Genotype_X)               .......... .......... .......... .......... ..S....... .......... .......... .......... .......... 450
KAB/62_(Genotype_XI)                  ---------- ---------- ---------- ---------- ---------- ---------- ---------- ---------- ---------- –
MZI/921_(Genotype_XII)                ---------- ---------- ---------- ---------- ---------- ---------- ---------- ---------- ---------- –
SUM/1411_(Genotype_XIII)              ---------- ---------- ---------- ---------- ---------- ---------- ---------- ---------- ---------- –
NYA/12_(Genotype_XIV)                 ---------- ---------- ---------- ---------- ---------- ---------- ---------- ---------- ---------- –
TAN/08/Mazimbu_(Genotype_XV)          .......... .....V.... .......... .......... .......... .......... .......... .......... .......... 450
TAN/2003/2_(Genotype_XVI)             ---------- ---------- ---------- ---------- ---------- ---------- ---------- ---------- ---------- –
ZIM/92/1_(Genotype_XVII)              ---------- ---------- ---------- ---------- ---------- ---------- ---------- ---------- ---------- –
NAM/1/95_(Genotype_XVIII)             ---------- ---------- ---------- ---------- ---------- ---------- ---------- ---------- ---------- –
SPEC/251_(Genotype_XIX)               ---------- ---------- ---------- ---------- ---------- ---------- ---------- ---------- ---------- –
Pretoriuskop/96/4_(Genotype_XX)       .......... .....V.... ..A....... .......... .......... .......... .......... .......... .......... 450
RSA/1/96_(Genotype_XXI)               ---------- ---------- ---------- ---------- ---------- ---------- ---------- ---------- ---------- –
SPEC/245_(Genotype_XXII)              ---------- ---------- ---------- ---------- ---------- ---------- ---------- ---------- ---------- –
ETH/017_(Genotype_XXIIIa)             .......... .......... .......... .......... ..S....... .......... .......... .......... .......... 450
ETH/AA_(Genotype_XXIIIb)              .......... .......... .......... .......... ..S....... .......... .......... .......... .......... 450
MOZ_10/2006_(Genotype_XXIV)           ---------- ---------- ---------- ---------- ---------- ---------- ---------- ---------- ---------- –
Conservation

                                      460                   480                   500                   520                   540
Benin_97/1_(Genotype_I)               HDEKLMSALK WPIEYMFIGL KPTWNISDQN PHQHRDWHKF GHVVNAIMQP THHAEISFQD RDTALPDACS SISDISPVTY PITLPIIKNI 540
ASFV_Georgia_2007/1_(Genotype_II)     .......... .......... .......... .......... .......... .......... .......... .......... .......... 540
Warmbaths_(Genotype_III)              .......... .......... .......... .......... .......... .......... .......... .......... .......... 540
Warthog_(Genotype_IV)                 .......... .......... .......... .......... .......... .......... .......... .......I.. .......... 540
Tengani_62_(Genotype_V)               .......... .......... .......... .......... .......... .......... .......... .......... .......... 540
MOZ/94/1_(Genotype_VI)                ---------- ---------- ---------- ---------- -------... .......... .......... .......... .......... 43
Mkuzi_1979_(Genotype_VII)             .......... .......... .......... .......... .......... .......... .......... .......... .......... 540
Malawi_Lil-20/1_(Genotype_VIII)       .......... .......... .......... .......... .......... .......... .......... .....N.... .......... 540
Ken06.Bus_(Genotype_IX)               .......... .......... .......... .......... .......... .....V.... .......... .....T.I.. .......... 540
Kenya_1950_(Genotype_X)               .......... .......... .......... .......... .......... .....V.... .......... .......I.. .......... 540
KAB/62_(Genotype_XI)                  ---------- ---------- ---------- ---------- -------... .......... .......... .....N.... .......... 43
MZI/921_(Genotype_XII)                ---------- ---------- ---------- ---------- -------... .......... .......... .....N.... .......... 43
SUM/1411_(Genotype_XIII)              ---------- ---------- ---------- ---------- -------... .......... .......... .....N.... .......... 43
NYA/12_(Genotype_XIV)                 ---------- ---------- ---------- ---------- -------... .......... .......... .....N.... .......... 43
TAN/08/Mazimbu_(Genotype_XV)          .......... .......... .......... .......... .......... .......... .......... .....N.... .......... 540
TAN/2003/2_(Genotype_XVI)             ---------- ---------- ---------- ---------- -------... .......... .......... .....N.... .......... 43
ZIM/92/1_(Genotype_XVII)              ---------- ---------- ---------- ---------- --------.. .......... .......... .......I.. .......... 42
NAM/1/95_(Genotype_XVIII)             ---------- ---------- ---------- ---------- --------.. .......... .......... .......... .......... 42
SPEC/251_(Genotype_XIX)               ---------- ---------- ---------- ---------- --------.. .......... .......... .......... .......... 42
Pretoriuskop/96/4_(Genotype_XX)       .......... .......... .......... .......... .......... .......... .......... .......... .......... 540
RSA/1/96_(Genotype_XXI)               ---------- ---------- ---------- ---------- --------.L .......... .......... .......I.. .......... 42
SPEC/245_(Genotype_XXII)              ---------- ---------- ---------- ---------- --------.. .......... .......... .......... .......... 42
ETH/017_(Genotype_XXIIIa)             .......... .......... .......... .......... .......... .....V.... .......... .....T.I.. .......... 540
ETH/AA_(Genotype_XXIIIb)              .......... .......... .......... .......... .......... .....V.... .......... .....T.I.. .......... 540
MOZ_10/2006_(Genotype_XXIV)           ---------- ---------- ---------- ---------- --------.. .......... .......... .......I.. .......... 42
Conservation
```

```
                                                       560                 580                 600                 620
                                                         |                   |                   |                   |
Benin_97/1_(Genotype_I)             SVTAHGINLI DKFPSKFCSS YIPFHYGGNA IKTPDDPGAM MITFALKPRE EYQPSGHINV SRAREFYISW DTDYVGSITT ADLVVSASAI 630
ASFV_Georgia_2007/1_(Genotype_II)   .......... .......... .......... .......... .......... .......... .......... .......... .......... 630
Warmbaths_(Genotype_III)            .......... .......... .......... .......... .......... .......... .......... .......... .......... 630
Warthog_(Genotype_IV)               .......... .......... .......... .......... .......... .......... .......... .......... .......... 630
Tengani_62_(Genotype_V)             .......... .......... .......... .......... .......... .......... .......... .......... .......... 630
MOZ/94/1_(Genotype_VI)              .......... .......... .......... .......... .......... .......... .......... .......... .......... 133
Mkuzi_1979_(Genotype_VII)           .......... .......... ........S. .......... .......... .......... .......... .......... .......... 630
Malawi_Lil-20/1_(Genotype_VIII)     .......... .......... .........S .......... .......... .......... .......... .......... .......... 630
Ken06.Bus_(Genotype_IX)             .......... .......... .........S .......... .......... .......... .......... .......... .......... 630
Kenya_1950_(Genotype_X)             .......... .......... .........S .......... .......... .......... .......... .......... .......... 630
KAB/62_(Genotype_XI)                .......... .......... .........S .......... .......... .......... .......... .......... .......... 133
MZI/921_(Genotype_XII)              .......... .......... .........S .......... .......... .......... .......... .......... .......... 133
SUM/1411_(Genotype_XIII)            .......... .......... .........S .......... .......... .......... .......... .......... ....ACG... 133
NYA/12_(Genotype_XIV)               .......... .......... .........S .......... .......... .......... .......... .......... .......... 133
TAN/08/Mazimbu_(Genotype_XV)        .......... .......... .........S .......... .......... .......... .......... .......... .......... 630
TAN/2003/2_(Genotype_XVI)           .......... .......... .........S .......... .......... .......... .......... .......... .......... 133
ZIM/92/1_(Genotype_XVII)            .......... .......... .......... .......... .......... .......... .......... .......... .......... 132
NAM/1/95_(Genotype_XVIII)           .......... .......... .......... .......... .......... .......... .......... .......... .......... 132
SPEC/251_(Genotype_XIX)             .......... .......... .......... .......... .......... .......... .......... .......... .......... 132
Pretoriuskop/96/4_(Genotype_XX)     .......... .......... .......... .......... .......... .......... .......... .......... .......... 630
RSA/1/96_(Genotype_XXI)             .......... .......... .......... .......... .......... .......... .......... .......... .......... 132
SPEC/245_(Genotype_XXII)            .......... .......... ........S. .......... .......... .......... .......... .......... .......... 132
ETH/017_(Genotype_XXIIIa)           .......... .......... .........S .......... .......... .......... .......... ..E....... .......... 630
ETH/AA_(Genotype_XXIIIb)            .......... .......... .........S .......... .......... .......... .......... ..E....... .......... 630
MOZ_10/2006_(Genotype_XXIV)         .......... .......... .......... .......... .......... .......... .......... .......... .......... 132
Conservation

                                             640
                                              |
Benin_97/1_(Genotype_I)             NFLLLQNGSA VLRYST* 647
ASFV_Georgia_2007/1_(Genotype_II)   .......... ....... 647
Warmbaths_(Genotype_III)            .......... ....... 647
Warthog_(Genotype_IV)               .......... ....... 647
Tengani_62_(Genotype_V)             .......... ....... 647
MOZ/94/1_(Genotype_VI)              ......---- ------- 139
Mkuzi_1979_(Genotype_VII)           .......... ....... 647
Malawi_Lil-20/1_(Genotype_VIII)     .......... ....... 647
Ken06.Bus_(Genotype_IX)             .......... ....... 647
Kenya_1950_(Genotype_X)             .......... ....... 647
KAB/62_(Genotype_XI)                ......---- ------- 139
MZI/921_(Genotype_XII)              ......---- ------- 139
SUM/1411_(Genotype_XIII)            ......---- ------- 139
NYA/12_(Genotype_XIV)               ......---- ------- 139
TAN/08/Mazimbu_(Genotype_XV)        .......... ....... 647
TAN/2003/2_(Genotype_XVI)           ......---- ------- 139
ZIM/92/1_(Genotype_XVII)            .....----- ------- 137
NAM/1/95_(Genotype_XVIII)           .....----- ------- 137
SPEC/251_(Genotype_XIX)             .....----- ------- 137
Pretoriuskop/96/4_(Genotype_XX)     .......... ....... 647
RSA/1/96_(Genotype_XXI)             .....----- ------- 137
SPEC/245_(Genotype_XXII)            .....----- ------- 137
ETH/017_(Genotype_XXIIIa)           .......... ....... 647
ETH/AA_(Genotype_XXIIIb)            .......... ....... 647
MOZ_10/2006_(Genotype_XXIV)         .--------- ------- 133
Conservation
```
