# Supplementary material for: A Re-Evaluation of African Swine Fever Genotypes Based on p72 Sequences Reveals the Existence of Only Six Distinct p72 Groups
Source: Viruses. 2023 Nov 11;15(11):2246. doi: 10.3390/v15112246 (PMC10675559; doi:10.3390/v15112246)

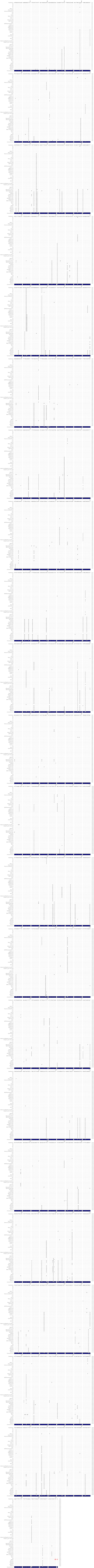

Supplement: Supplementary file 1 [file Figure S3 Alignment of 55 full-length P72 coding DNA sequences.pdf › Figure S3 Alignment of 55 full-length P72 coding DNA sequences.pdf]
